# Safety and immunogenicity of investigational tuberculosis vaccine M72/AS01_E–4_ in people living with HIV in South Africa: an observer-blinded, randomised, controlled, phase 2 trial

**DOI:** 10.1016/S2352-3018(25)00124-9

**Published:** 2025-07-01

**Authors:** Alemnew F Dagnew, Linda L Han, Kogieleum Naidoo, Lee Fairlie, James C Innes, Keren Middelkoop, Michele Tameris, Robert J Wilkinson, Jintanat Ananworanich, Daniel Bower, Lisa Schlehuber, Nicole Frahm, Amy Cinar, Michael Dunne, Alexander C Schmidt

**Affiliations:** a Gates Medical Research Institute, Clinical Development, Cambridge, MA, USA; b CAPRISA-South African Medical Research Council (SAMRC) TB-HIV Pathogenesis and Treatment Research Unit, University of KwaZulu-Natal Nelson R Mandela School of Medicine, Durban, South Africa; c Wits RHI, Faculty of Health Sciences, University of the Witwatersrand, Johannesburg, South Africa; d The Aurum Institute, Clinical Research Division, Aurum Klerksdorp Clinical Research, Klerksdorp, South Africa; e Desmond Tutu HIV Center, Institute of Infectious Disease and Molecular Medicine, Department of Medicine, University of Cape Town, Cape Town, South Africa; f South African Tuberculosis Vaccine Initiative (SATVI), Department of Pathology and Institute of Infectious Disease and Molecular Medicine, University of Cape Town, Cape Town, South Africa; g Wellcome Discovery Research Platforms in Infection, Center for Infectious Diseases Research in Africa, Institute of Infectious Disease and Molecular Medicine and Department of Medicine, University of Cape Town, Cape Town, South Africa; h Francis Crick Institute, London, UK; i Department of Infectious Diseases, Imperial College London, London, UK

## Abstract

**Background:**

M72/AS01_E–4_ is a recombinant fusion protein vaccine candidate derived from two *Mycobacterium tuberculosis* antigens (*Mtb*32A and *Mtb*39A) and AS01_E–4_ adjuvant. We evaluated safety and immunogenicity of M72/AS01_E–4_ in people living with HIV in South Africa.

**Methods:**

In this observer-blinded, randomised, controlled, phase 2 trial, participants aged 16–35 years with well controlled HIV were enrolled from urban, semi-urban, and semi-rural settings in South Africa, including sites with high tuberculosis and HIV prevalence, as well as agricultural and mining communities. Participants were randomly assigned (1:1), stratified by site and interferon-gamma release assay (IGRA) status, to receive two intramuscular doses of M72/AS01_E–4_ or placebo. Eligibility criteria included antiretroviral therapy for at least 3 months, HIV viral load of less than 200 copies per mL, CD4 counts of 200 cells per μL or higher, and previous completion of tuberculosis preventive therapy and no tuberculosis history. The sponsor and its delegates, the laboratory team, investigators, site staff, and participants were blinded to randomisation, whereas an unblinded pharmacist who was not involved in trial procedures prepared placebo and reconstituted M72/AS01_E-4_ in unit-dose syringes covered with a blinding label. All participants who received at least one dose of either M72/AS01_E–4_ or placebo were included in the safety population for safety analyses. Immunogenicity analyses were conducted using the per-protocol population, which included participants who received the intervention as planned and did not substantially deviate from the protocol procedures. Safety assessments included solicited adverse events in the first 7 days after each dose, unsolicited adverse events in the first 28 days after each dose, and serious adverse events. Humoral responses were measured with ELISA and cellular responses were assessed using multiparameter flow cytometry, in the per-protocol population. This study is complete and is registered with ClinicalTrials.gov, NCT04556981.

**Findings:**

Between Nov 17, 2020, and Aug 12, 2022, 402 eligible participants were assigned treatment, of whom 401 participants received at least one dose of M72/AS01_E–4_ (n=201; 175 [87%] were female and 26 [13%] were male; 196 [98%] were Black) or placebo (n=200; [176 [88%] were female and 24 [12%] were male; 196 [98%] were Black) and followed for a median duration of 372 days (IQR 364–389). Among M72/AS01_E–4_ recipients, solicited adverse events were more frequent, ranging from 17% (33 of 199) for gastrointestinal symptoms to 77% (140 of 183) for injection-site pain. Most events were mild to moderate, with severe events ranging from 0% (0 of 197) for swelling and (0 of 198) redness to 13% (24 of 183) for injection-site pain, resolving within 3 days. Unsolicited adverse events related to vaccine were mainly injection-site reactions in the M72/AS01_E–4_ group (8% [15 of 201] *vs* 1% [two of 200] in the placebo group), including erythema, pruritis, swelling, bruising, induration, and pain. No vaccine-related serious adverse events were reported. Among M72/AS01_E–4_ recipients at day 57 (1 month after dose two), M72-specific antibody geometric mean concentration (GMC) was 479·70 EU/mL (95% CI 421·79–545·56) with median magnitude of CD4 cells of 0·383% (IQR 0·177%–0·663). Among M72/AS01_E–4_ recipients, at day 57 GMCs were 559·49 EU/mL (95% CI 461·75–677·93) in with baseline IGRA positivity and 424·95 EU/mL (357·74–504·80) in those without; median magnitudes of CD4 cells were 0·447% (IQR 0·287–0·819) and 0·321% (0·147–0·581).

**Interpretation:**

The two-dose regimen of the M72/AS01_E–4_ tuberculosis vaccine was immunogenic, with an acceptable safety profile. These outcomes led to the inclusion of people living with HIV in the ongoing global registration phase 3 trial.

**Funding:**

Gates Foundation and the Wellcome Trust.

## Introduction

In 2022, an estimated 10·6 million people fell ill with tuberculosis and an estimated 1·3 million died, including 167 000 people living with HIV,.^1^ HIV infection has a major impact on host cell-mediated responses to *Mycobacterium tuberculosis* and is a risk factor for tuberculosis.^2^ People living with HIV who are infected with *M tuberculosis* are more likely to progress to tuberculosis than are people not infected with HIV.^2^ Tuberculosis might also affect immune responses to HIV and accelerate progression to AIDS.^3^ The proportion of people living with HIV with a new diagnosis of tuberculosis was highest in the WHO African Region, with parts of southern Africa exceeding 50%.^1^

**Research in context**

**Evidence before this study**

BCG is the only licensed tuberculosis vaccine. However, its effect on the transmission of *Mycobacterium tuberculosis* is limited, as it provides little protection against pulmonary tuberculosis in adults or adolescents. Therefore, the development of a new vaccine with an acceptable safety profile that prevents pulmonary tuberculosis and could be administered regardless of HIV status and interferon-gamma release assay (IGRA) status would be a notable advance in the global effort to end the tuberculosis epidemic. The investigational M72 tuberculosis vaccine formulated with AS01_E–4_ (M72/AS01_E–4_ vaccine) has been evaluated in a series of clinical trials over years, culminating in a phase 2b trial demonstrating 49·7% vaccine efficacy against prevention of pulmonary tuberculosis in approximately 3500 adults with tuberculosis infection who were HIV negative. Between Dec 1 and Dec 6, 2024, we conducted a search for relevant studies in PubMed without language restrictions using the following combination of terms: (“M72/AS01” OR “M72” OR “AS01”) AND (“South Africa” OR “Republic of South Africa”) AND (“Human immunodeficiency virus” OR “HIV” OR “People Living with HIV” OR “PLHIV”). The search identified publications; however, none of them focused on the M72/AS01_E–4_ investigational vaccine study in people living with HIV in the same population setting.

**Added value of this study**

This observer-blinded, randomised, controlled, phase 2 trial in South Africa expands on the existing safety and immunogenicity data from two earlier trials of the M72/AS01_E–4_ vaccine in people living with HIV, focusing on a larger trial population of people living with HIV residing in a country with a high burden of tuberculosis. The M72/AS01_E–4_ vaccine was well tolerated with an acceptable safety profile, and led to robust humoral and CD4 cell responses. In addition, this trial included participants with either positive or negative IGRA results at baseline. Immunogenicity analysis demonstrated booster effects for antibody responses in participants with IGRA-positive status and priming in participants with IGRA-negative status.

**Implications of all the available evidence**

The safety and immunogenicity data in this trial were consistent with previous M72/AS01_E–4_ clinical trials and included a large population of people living with HIV in a country with a high burden of tuberculosis. This trial provided supportive data that led to the inclusion of people living with HIV in a M72/AS01_E–4_ global registration phase 3 trial.

Antiretroviral therapy (ART) is the most effective preventive strategy against tuberculosis for people living with HIV, substantially reducing the risk of tuberculosis irrespective of baseline CD4 cell counts, *M tuberculosis* infection, and tuberculosis drug resistance.^4^, ^5^ However, ART alone cannot control tuberculosis. ART is often started late during HIV progression and many people living with HIV already have tuberculosis before starting ART. BCG is the only licensed tuberculosis vaccine; however, it has limited impact on transmission of *M tuberculosis* because it offers minimal or no protection against pulmonary tuberculosis in adults or adolescents.^6^ Considering the substantial morbidity and mortality associated with tuberculosis, a new vaccine is urgently needed to protect individuals and to expedite the end of the tuberculosis epidemic.

The investigational vaccine M72/AS01_E–4_ (GlaxoSmithKline, Wavre, Belgium) has been in development since the early 2000s, with the intent to prevent tuberculosis in adolescents and adults. The vaccine comprises a recombinant fusion protein (M72) produced in *Escherichia coli*, derived from two immunogenic *M tuberculosis* antigens (*Mtb*32A and *Mtb*39A), combined with the GlaxoSmithKline proprietary adjuvant system AS01_E–4_.^7^ *Mtb*32A is a secreted putative serine protease, and *Mtb*39A is a membrane-associated, putative evasion factor that belongs to the proline-proline-glutamic acid family of proteins found in mycobacteria.^8^ In individuals with latent or active tuberculosis, these antigens can induce specific lymphoproliferative responses and interferon (IFN)-γ production.^9^, ^10^, ^11^

M72/AS01_E–4_ was evaluated in early clinical trials, which included a range of populations and settings, including two small trials in people living with HIV conducted in Switzerland (phase 1/2)^12^ and India (phase 2).^13^, ^14^ In these trials, 102 people living with HIV, 62 of whom were virally suppressed on ART, who had no past or current tuberculosis disease received M72/AS01_E_ intramuscularly. In these trials, the vaccine induced cell-mediated and humoral responses that persisted up to 3 years.^14^ No safety concerns were raised, and there were no clinically significant effects on participants' HIV-1 viral loads, CD4-cell counts, or ART regimens.^13^, ^14^

The M72/AS01_E–4_ phase 2b vaccine trial, conducted in three tuberculosis-endemic countries in Africa (Kenya, South Africa, and Zambia), reported safety and efficacy in approximately 3500 HIV-negative adults with *M tuberculosis* infection, based on positive interferon-gamma release assay (IGRA) results.^7^ Observed vaccine efficacy against laboratory-confirmed pulmonary tuberculosis was 49·7% (95% CI 2·1–74·2) after 36 months follow-up, with an acceptable safety profile.^7^ These positive outcomes led to a large phase 3 registrational efficacy trial (Gates Medical Research Institute; NCT06062238), which is ongoing. The goal of this trial was to expand on existing data, focusing on a larger group of people living with HIV in South Africa, a country with a high burden of tuberculosis and HIV, and to support potential inclusion of people living with HIV in the phase 3 M72/AS01_E–4_ vaccine trial.

## Methods

### Study design and participants

We conducted an observer-blinded, phase 2, randomised, controlled, phase 2 trial to evaluate safety and immunogenicity of two doses (given 1 month apart) of M72/AS01_E–4_ tuberculosis vaccine versus placebo. The clinical trial is registered at ClinicalTrials.gov, NCT04556981, and in the South African National Clinical Trials Registry, DOH-27–102020–6567. Participants were followed up for 12 months after dose two. Participants were aged 16–35 years, living with well controlled HIV who were virally suppressed (HIV viral load <200 copies per mL) on ART. Participants were recruited from urban, semi-urban, and semi-rural settings, including sites with high tuberculosis and HIV rates, and sites beyond urban centres where agriculture and mining were common. Participants provided written consent (or assent if applicable) and were enrolled at six clinical sites in Cape Town, Worcester, Durban, Johannesburg, and Klerksdorp in South Africa. Participants were enrolled when they were on ART for at least 3 months, had HIV viral load of less than 200 copies per mL, had CD4 counts of 200 cells per μL or higher, had previously completed a course of tuberculosis preventive therapy, and had no tuberculosis history. Additional details about eligibility criteria are in the appendix (p 5).

The trial was done in accordance with Good Clinical Practice guidelines applicable at the time and the Declaration of Helsinki. The ethics committees that reviewed and approved the study were the University of Capetown Human Research Ethics Committee (registration number REC-210208–007), the Biomedical Research Ethics Committee, University of KwaZulu Natal (registration number REC-290408–009), and the University of the Witwatersrand (registration number REC-250208–004). The protocol is available on online.

### Randomisation and masking

Participants were randomly assigned (1:1) on day 1, to either the M72/AS01_E–4_ group or the placebo group, using an interactive web randomisation system integrated into the Zelta Electronic Data Capture (EDC) system. Randomisation was stratified by site and by baseline IGRA status (positive or negative); an indeterminate IGRA test result at baseline rendered the participant ineligible. Block randomisation with size four was used to balance enrolment into each group. Study pharmacists at each trial site were granted secure unmasked access to the randomisation module within the EDC system.

The sponsor and its delegates, the laboratory team, investigators, site staff, and participants were masked to randomisation. However, due to the different appearances of M72/AS01_E–4_ and placebo, the trial was conducted in an observer-blinded manner. To maintain a double-blind trial, the unmasked pharmacist, who was uninvolved in trial procedures, provided placebo and reconstituted M72/AS01_E–4_ as unit-dose syringes covered with a blinding label. Designated trial monitors, the Independent Data Monitoring Committee members, and the biostatistician who prepared the randomisation list and statistical outputs were all unmasked.

### Procedures

Each participant was to receive two doses of either M72/AS01_E–4_ investigational tuberculosis vaccine or placebo, injected in the deltoid muscle on day 1 and day 29. Each 0·5 mL dose of M72/AS01_E–4_ contained 10 μg M72 recombinant fusion protein reconstituted with the AS01_E–4_ adjuvant system containing three components: 25 μg MPL (3-O-desacyl-4-monophosphoryl lipid A produced by GlaxoSmithKline (Hamilton, MT, USA)), 25 μg QS-21 (*Quillaja saponaria* Molina, fraction 21; licensed by GlaxoSmithKline from Antigenics, a wholly owned subsidiary of Agenus, Lexington, MA, USA), and liposomes. The M72/AS01_E–4_ reconstituted vaccine is a homogeneous, opalescent liquid. The placebo was a 0·5 mL dose of normal saline (0·9% NaCl solution; Adcock Ingram Critical Care, Johannesburg, South Africa).

No dose reductions or interruptions were allowed in the trial. Participants who withdrew from the trial due to a serious adverse event or an adverse event were followed up by investigators until resolution or stabilisation of the event. Data on any treatments received after stopping the trial intervention were not required by the study protocol and not collected.

To evaluate eligibility criteria, testing was performed centrally at Bio Analytical Research Corporation, Johannesburg, South Africa (appendix p 5). For humoral immune responses, we measured M72-specific IgG antibody serum concentrations using a custom direct ELISA previously developed at The Center for Vaccinology (University of Ghent, Ghent, Belgium).^15^ Positivity was defined as M72-specific antibody concentrations of 2·8 EU/mL or higher, the lower limit of quantification. For cell-mediated immune responses, cryopreserved peripheral blood mononuclear cells (PBMCs) were analysed at Precision for Medicine (Frederick, MD, USA) by eight-colour intracellular cytokine staining flow cytometry, with dimethyl sulfoxide (DMSO) as the negative control or background, and a peptide pool covering the M72 antigen (178 overlapping peptides of 15 amino acids in length) to assess antigen-specific responses (appendix pp 6–9).

Active surveillance for signs and symptoms compatible with incident tuberculosis disease (cough, fever, fatigue, or night sweats, for longer than 2 weeks, or loss of weight) was conducted at each visit throughout the trial. Evidence of signs or symptoms led to sputum sample collection and an Xpert MTB/RIF assay (Cepheid, Sunnyvale, California, USA**)**. Data on self-reported sex and race were collected.

### Outcomes

The primary trial objective was to assess safety and reactogenicity of M72/AS01_E–4_ vaccination in people living with HIV. Endpoints were solicited adverse events (reactogenicity) up to 7 days after each dose, unsolicited adverse events up to 28 days after each dose, and serious adverse events throughout the trial. We used the US National Institutes of Health Division of Allergies and Infectious Diseases (DAIDS) toxicity table^16^ to grade adverse events.

Solicited local adverse events were pain, redness, and swelling at injection site, and systemic adverse events were headache, fatigue, myalgia, gastrointestinal symptoms, and fever. Data were recorded on paper diary cards by each participant for 7 days after each dose. Unsolicited adverse events were collected for the first 28 days after each dose. Solicited adverse events that continued beyond day 7, as well as verbally reported solicited adverse events, were counted as unsolicited adverse events. All serious adverse events, potential immune-mediated diseases (pIMDs), and pregnancies were recorded from day 1 to month 13 (day 390, end of trial). Unsolicited adverse events were coded with MedDRA version 23.1 and pIMDs were identified with prespecified MedDRA PT codes. Secondary safety endpoints were pIMDs and laboratory assessments (haematology and serum chemistry) defined as grade 3 (severe) or higher (worse).^16^

Other secondary endpoints were the characterisation of M72-specific IgG antibody concentration and seropositivity on days 1, 29, 57, 210, and 390, and the percentage of participants with post-baseline M72-specific CD4 and CD8 cell responses and the magnitude of those responses (all DMSO background-subtracted). The magnitude of M72/AS01_E–4_-specific CD4 and CD8 T-cell response is the percentage of these cells expressing IFN-γ or IL-2 in response to stimulation with M72 peptides. Exploratory endpoints included the polyfunctionality of post-baseline M72-specific T-cell responses as measured by expression of each combination of the functional markers: CD40L (CD4 cells only), IFN-γ, IL-2, and tumour necrosis factor (appendix p 31).

### Statistical analysis

No formal statistical hypotheses were tested. However, with 200 participants vaccinated with M72/AS01_E–4_, there was at least 90% power to observe at least one adverse event if the true rate was at least 1·2% (80% if the true rate was at least 0·8%). Thus, it was highly probable to observe at least one serious adverse event, or one pIMD even if the true adverse event rate was relatively low (~1%).

All participants who received at least one dose of either M72/AS01_E–4_ or placebo were included in the safety population for safety analyses, and assessment of suspected tuberculosis cases. Participants who received intervention as planned and did not substantially deviate from the protocol procedures (ie, the per-protocol population) were included in humoral immunogenicity assessments. The per-protocol population for cellular immunogenicity included participants from the per-protocol population who were randomly selected based on treatment status (3:1 treatment *vs* placebo) and sample availability (had at least one vial of PBMCs at days 1, 57, and 390 with ≥8 million PBMCs per vial).

The number and percentage of participants with solicited adverse events, unsolicited adverse events, serious adverse events, pIMDs, HIV viral load of less than 200 copies per mL, and CD4 counts of less than 350 cells per μL were assessed during the applicable timepoints, per group. For each assessment, the Clopper–Pearson method with mid-p correction was used in the calculation of the 95% CI for the proportion within a group and timepoint. The p values for HIV viral load and CD4 cell counts were based on Fisher's exact test comparing the proportion of participants between groups. The median duration (IQR) of each solicited adverse event was summarised.

To assess humoral immunogenicity, individual M72-specific IgG antibody concentrations were log-transformed. Geometric mean concentrations (GMCs) with 95% CIs were summarised per group and timepoint, by exponential transformation of the log-transformed concentration data and its 95% CI (based on the *t*-distribution). Seropositivity percentage per group and timepoint were calculated with a cutoff value for M72-specific antibody concentrations of 2·8 EU/mL. The 95% CIs for a proportion within a group and timepoint were based on the mid-p method.

Magnitude of post-baseline M72-specific CD4 and CD8 cell responses (DMSO-subtracted), per visit, were summarised and compared between groups by use of Wilcoxon–Mann–Whitney test for pairwise comparisons. Magnitude of M72-specific CD4 cell responses was summarised as median (IQR). The number and percentage of participants with post-baseline M72-specific CD4 and CD8 cell responses were summarised, including a comparison of the groups, by use of Fisher's exact test. For each timepoint for each participant, a positive response was defined as a positive CD4 cell IFN-γ or IL-2 value post-DMSO subtraction. Subsequent overall individual responder status was determined by an algorithm (appendix p 10). When both baseline and post-baseline values were positive post-DMSO subtraction, then the Breslow–Day test and odds ratios (ORs) were used to determine overall individual responder status by comparing baseline and post-baseline number of CD4 cells expressing IFN-γ or IL-2. The same approach was taken for the calculation of positive M72 CD8^+^ T-cell responses. Missing immunogenicity values were considered missing completely at random and therefore were not considered to contain information that affected the result of the analysis (ie, not informative). Imputation methods were therefore not used. Participants who were missing baseline or post-baseline values were excluded from change from baseline analyses.

Analyses were also performed based on IGRA status at baseline, to determine the effect of *M tuberculosis* infection with respect to humoral and cellular immunogenicity, and reactogenicity. Analyses were performed in SAS version 9.4. A p value threshold of 0·05 was used.

### Role of the funding source

The funders of the study had no role in study design, data collection, data analysis, data interpretation, or writing of the report.

## Results

**Figure 1:**
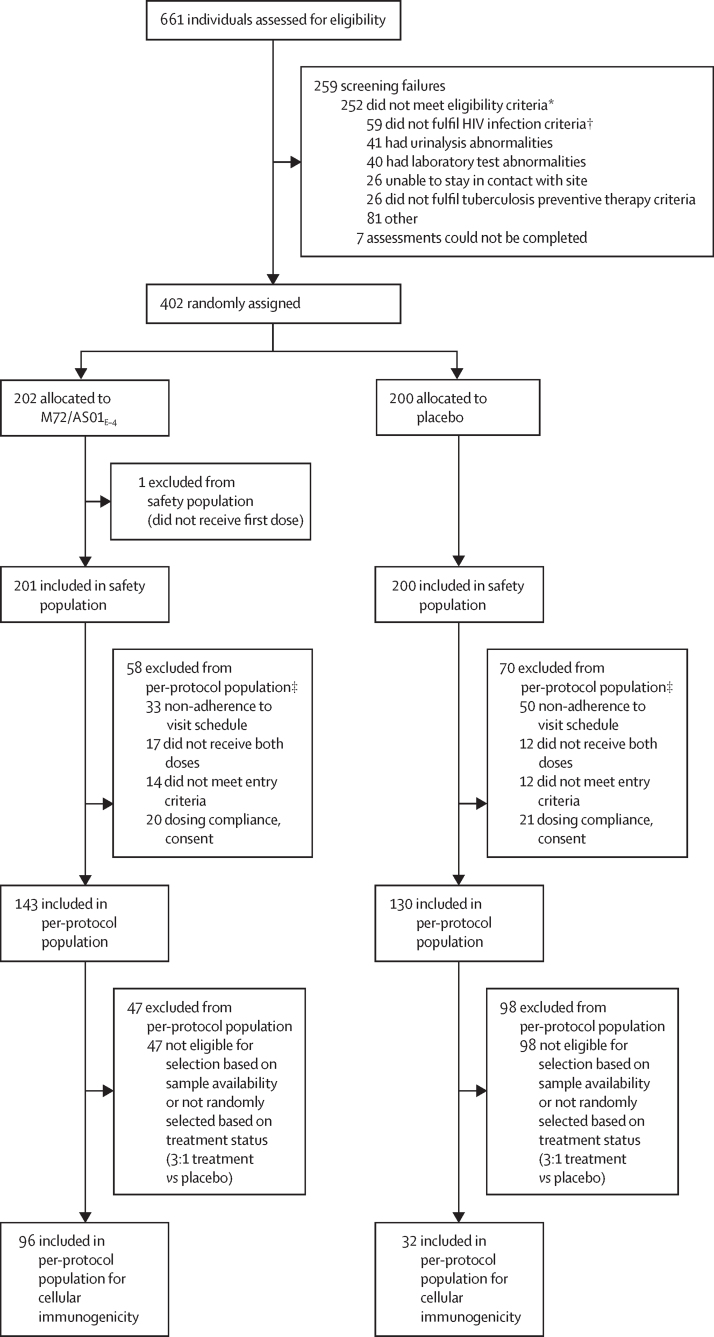
Trial profile *People might have had more than one reason for ineligibility. †40 had HIV viral loads ≥200 copies per mL, eight had CD4 counts <200 cells per μL and HIV viral loads ≥200 copies per mL, five did not have at least 3 consecutive months on antiretroviral therapy, five had CD4 counts <200 cells per μL, and one did not have a reactive anti-HIV antibody. ‡People might have had more than one reason for exclusion from per-protocol population.

The trial was conducted from Nov 17, 2020, to Aug 12, 2022. Of 661 individuals assessed for eligibility, 259 did not meet all eligibility criteria. 402 participants were randomly assigned across the six trial sites. Of these, 401 received the first dose of M72/AS01_E–4_ (n=201) or placebo (n=200) and were included in the safety population (figure 1).

373 (93%) participants in the safety population received both doses: 185 (92%) in the M72/AS01_E–4_ group and 188 (94%) in the placebo group. 376 (94%) participants completed the trial: 195 (97%) in the M72/AS01_E–4_ group and 181 (91%) in the placebo group. The primary reason for trial discontinuation was loss to follow-up. The per-protocol population included 273 (68%) participants (appendix p 11), and the per-protocol population for cellular immunogenicity included 128 (32%) participants. Participants were distributed similarly across groups in terms of demographic and key baseline characteristics (table 1).

**Table 1:** Summary of demographic data and key baseline characteristics in the safety population

		**M72/AS01**_E–4_**(n=201)**	**Placebo (n=200)**
Age, years		29·4 (4·17)	29·6 (4·47)
Sex assigned at birth			
	Male	26 (13%)	24 (12%)
	Female	175 (87%)	176 (88%)
Race			
	Black	196 (98%)	196 (98%)
	South African Coloured	5 (3%)	4 (2%)
IGRA-positive at baseline		93 (46%)	100 (50%)
IGRA-negative at baseline		108 (54%)	100 (50%)
Weight, kg		73·4 (19·93)	74·3 (18·81)
Height, cm		161·8 (7·20)	161·5 (7·70)
BMI, kg/m^2^		28·1 (7·56)	28·5 (7·29)
CD4 count, cells per μL			
	200–349	7 (4%)	5 (3%)
	350–499	20 (10%)	24 (12%)
	≥ 500	174 (87%)	171 (86%)
HIV viral load, copies per mL			
	Not detected*	162 (81%)	162 (81%)
	≤ 200†	36 (18%)	35 (18%)
	> 200	3 (2%)†	3 (2%)†

Data are mean (SD) or n (%). Percentages calculated as number of participants in each trial group in the relevant analysis set with data available as the denominator. South African 'Colored’ refers to people of mixed race. IGRA=interferon-γ release assay.

* Samples for which HIV viral load was detectable but not quantifiable.

† Six participants (three per group) had HIV viral load ≤200 copies per mL at screening but had >200 copies per mL at day 1.

Any solicited adverse events were reported more frequently among participants who received M72/AS01_E–4_ vaccine than among those who received placebo (table 2), with the exception of gastrointestinal symptoms (appendix p 27). Injection-site pain, headache, and fatigue were the most common solicited adverse events after M72/AS01_E–4_ vaccine administration. Among vaccine recipients, most solicited adverse events were mild or moderate in intensity.

**Table 2:** Solicited adverse events by dose (safety population)

		**Dose one**		**Dose two**	
		M72AS01_E–4_ (n=201)	Placebo (n=200)	M72/AS01_E–4_ (n=185)	Placebo (n=188)
Number of participants experiencing any injection-site adverse events		n=201	n=197	n=184	n=187
Any injection-site adverse events		155 (77·1%; 70·9–82·5)	49 (24·9%; 19·2–31·3)	147 (79·9%; 73·6–85·2)	29 (15·5%; 10·8–21·2)
Pain		n=201	n=195	n=183	n=184
	Any	139 (69·2%; 62·5–75·2)	40 (20·5%; 15·3–26·6)	140 (76·5%; 70·0–82·2)	26 (14·1%; 9·6–19·7)
	Severe	10 (5·0%; 2·6–8·7)	1 (0·5%; 0·0–2·5)	24 (13·1%; 8·8–18·6)	1 (0·5%; 0·0–2·7)
Redness		n=198	n=196	n=178	n=186
	Any	46 (23·2%; 17·7–29·5)	17 (8·7%; 5·3–13·3)	45 (25·3%; 19·3–32·1)	7 (3·8%; 1·7–7·3)
	Severe	0 (0·0–0·02)	0 (0·0–0·02)	0 (0·0–0·02)	0
Swelling		n=197	n=196	n=181	n=187
	Any	62 (31·5%; 25·3–38·2)	11 (5·6%; 3·0–9·5)	61 (33·7%; 27·1–40·8)	8 (4·3%; 2·0–8·0)
	Severe	0 (0·0–0·02)	0 (0·0–0·02)	0 (0·0–0·02)	0 (0·0–0·02)
Number of participants experiencing any systemic adverse events		n=201	n=197	n=184	n=188
	Any systemic adverse events	132 (65·7%; 58·9–72·0)	109 (55·3%; 48·3–62·2)	134 (72·8%; 66·1–78·9)	83 (44·1%; 37·2–51·3)
	Fever	n=200	n=195	n=184	n=185
	Any	39 (19·5%; 14·4–25·4)	21 (10·8%; 7·0–15·7)	62 (33·7%; 27·1–40·8)	31 (16·8%; 11·9–22·7)
	Severe	2 (1·0%; 0·2–3·3)	1 (0·5%; 0·0–2·5)	1 (0·5%; 0·0–2·7)	1 (0·5%; 0·0–2·6)
Headache		n=200	n=196	n=183	n=188
	Any	92 (46·0%; 39·2–52·9)	61 (31·1%; 24·9–37·9)	104 (56·8%; 49·6–63·9)	44 (23·4%; 17·8–29·9)
	Severe	5 (2·5%; 0·9–5·5)	3 (1·5%; 0·4–4·1)	18 (9·8%; 6·1–14·8)	4 (2·1%; 0·7–5·1)
Fatigue		n=199	n=196	n=183	n=187
	Any	81 (40·7%; 34·0–47·6)	64 (32·7%; 26·4–39·5)	95 (51·9%; 44·7–59·1)	41 (21·9%; 16·4–28·3)
	Severe	3 (1·5%; 0·4–4·0)	2 (1·0%; 0·2–3·3)	11 (6·0%; 3·2–10·2)	2 (1·1%; 0·2–3·5)
Gastrointestinal symptoms		n=199	n=196	n=183	n=187
	Any	33 (16·6%; 11·9–22·2)	26 (13·3%; 9·0–18·6)	32 (17·5%; 12·5–23·5)	16 (8·6%; 5·1–13·2)
	Severe	1 (0·5%; 0·0–2·5)	0 (0·0–0·02)	3 (1·6%; 0·4–4·4)	0 (0·0–0·02)
Myalgia		n=199	n=196	n=183	n=188
	Any	58 (29·1%; 23·1–35·7)	35 (17·9%; 13·0–23·7)	73 (39·9%; 33·0–47·1)	22 (11·7%; 7·7–16·9)
	Severe	7 (3·5%; 1·6–6·8)	0 (0·0–0·02)	13 (7·1%; 4·0–11·5)	2 (1·1%; 0·2–3·5)

Data are n (%; 95% CI). Data are reported as collected on the diary card from days 1 to 7 after dose one and dose two. Denominators varied based on the number of participants who were vaccinated and had available diary data. Participants were categorised under the highest severity experienced during days 1 to 7 after dose one and dose two. 95% CIs of the percentages were computed using the Clopper–Pearson method with mid-p correction. Any injection-site adverse event included participants with pain, redness (>0 mm), or swelling (>0 mm). Any redness or swelling refers to diameter >0 mm; severe (grade 3) ≥100 mm. Any systemic adverse events included participants with any fever (≥37·5°C), headache, fatigue (tiredness), gastrointestinal symptoms (stomach problems), or myalgia (muscle pain). Any fever refers to temperature ≥37·5°C; severe fever refers to ≥39·3 to <40·0°C.

In the M72/AS01_E–4_ group, the percentages of participants with solicited adverse events (any grade and each grade) were largely similar between dose one and dose two, with the exception of any-grade fever, severe injection-site pain, and severe headache. In the M72/AS01_E–4_ group, median number of days of pain, redness, or swelling ranged from 2 days to 3 days after either dose, compared with a range of 1 day to 2 days in the placebo group (appendix pp 12–13). The median number of days of each of the systemic adverse events (1 day to 2 days) was similar across both groups after any dose.

The percentage of participants reporting unsolicited adverse events was similar between groups overall, and when categorised by intensity severe or worse (table 3). The most frequently reported was headache, reported by 18 (9%) of 201 participants in the M72/AS01_E–4_ group and 25 (13%) of 200 in the placebo group, followed by cough reported by ten (5%) of 201 participants versus six (3%) of 200. Other unsolicited adverse events reported by 4% of participants in the M72/AS01_E–4_ group included dizziness, injection-site erythema, myalgia, and upper respiratory tract infection. All other unsolicited adverse events were reported by 3% or less participants (appendix pp 14–15).

**Table 3:** Summary of unsolicited adverse events (safety population)

	**M72/AS01**_E–4_**(n=201)**		**Placebo (n=200)**	
	n (%)	95% CI	n (%)	95% CI
Unsolicited adverse events	94 (46·8%)	39·9–53·7	87 (43·5%)	36·7–50·4
Related unsolicited adverse events	25 (12·4%)	8·4–17·6	11 (5·5%)	2·9–9·4
Severe or worse unsolicited adverse events	3 (1·5%)	0·4–4·0	6 (3·0%)	1·2–6·1
Related–severe or worse unsolicited adverse events	2 (1·0%)	0·2–3·2	2 (1·0%)	0·2–3·3
Serious adverse event	4 (2·0%)	0·6–4·7	5 (2·5%)	0·9–5·5
Serious adverse event with outcome of death	0	..	1 (0·5%)	0·0–2·4
Potential immune-mediated disease	0	..	0	..

All unsolicited adverse events with onset on or after each dose and through the post-dosing reporting window of 28 days and with onset up to the end of trial (month 13) if these events were serious adverse events or potential immune-mediated diseases. Participants with multiple adverse events with different severities were counted only once under highest severity. Adverse event severity was categorised as mild (grade 1, not interfering with normal daily activities), moderate (grade 2, interfering with normal daily activities), severe (grade 3, preventing normal daily activities), or potentially life-threatening (grade 4). 95% CIs of the proportions were computed using the Clopperr–Pearson method with mid-p correction.

25 (12%) of 201 unsolicited adverse events were judged by investigators to be related to trial intervention in the M72/AS01_E–4_ group compared with 11 (6%) of 200 in the placebo group (appendix p 16). Among adverse events related to trial intervention, the difference in percentages of participants between the M72/AS01_E–4_ and the placebo groups was largely attributed to participants who had injection-site adverse events in the M72/AS01_E–4_ group (8% [15 of 201] *vs* 1% [two of 200] in the placebo group), including erythema, pruritis, swelling, bruising, induration, and pain. Of the reported severe unsolicited adverse events related to trial intervention, two were reported in the M72/AS01_E–4_ group (one participant with dizziness and one participant with injection-site erythema) and two in the placebo group (one participant with hypersensitivity and one participant with elevated aspartate aminotransferase). No grade 4-related unsolicited adverse events or pIMDs were reported. There were no premature trial discontinuations due to adverse events.

Overall, nine serious adverse events were reported for nine (2%) participants in the safety population: four in the M72/AS01_E–4_ group (COVID-19, tibia fracture, spontaneous abortion, and psychotic disorder) and five in the placebo group (COVID-19, anal abscess, spontaneous abortion, substance-induced psychotic disorder, and physical assault). One fatal serious adverse event was reported in the placebo group (physical assault). All serious adverse events were judged by investigators to be unrelated to trial intervention.

There were no clinically meaningful trends or differences in haematological (appendix p 17) or serum chemistry (appendix p 18) parameters between the trial groups. The percentages of participants with HIV viral loads of more than 200 copies per mL (appendix p 19) and the percentages of participants with CD4 counts less than 350 cells per μL (appendix p 20) were similar between groups at each timepoint. Seven participants reported signs or symptoms of tuberculosis, but none had laboratory-confirmed pulmonary tuberculosis. Pregnancy outcomes are described in the appendix (p 26).

**Figure 2:**
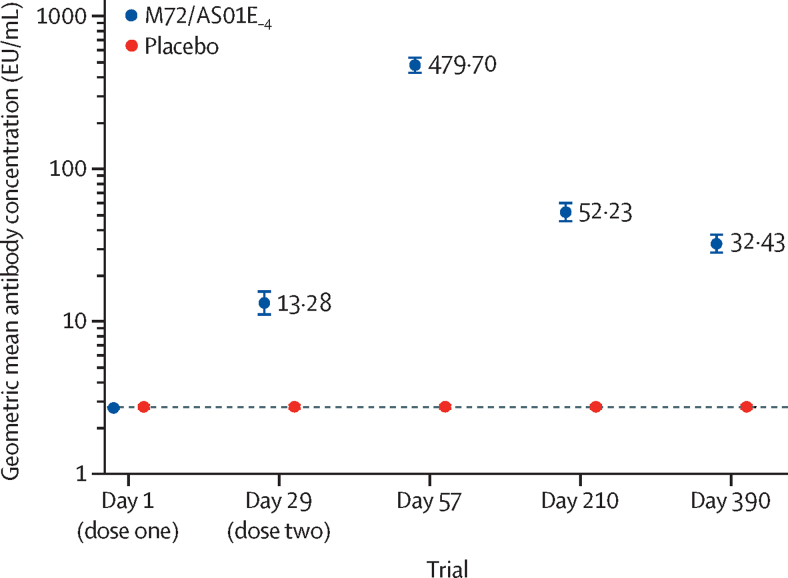
Geometric mean concentration for M72-specific antibodies (per-protocol population)

Two (1%) of 143 participants were seropositive for M72 antibody in the M72/AS01_E–4_ group at baseline, 128 (90%) of 143 were seropositive at day 29, and more than 98% were for the remaining timepoints. Four (3%) of 130 participants in the placebo group were seropositive at baseline, and this proportion remained constant at all timepoints (appendix p 21). GMCs of M72-specific antibodies in the M72/AS01_E–4_ and placebo groups were less than the assay cutoff of 2·8 EU/mL at baseline. In the M72/AS01_E–4_ group, GMC increased to 13·28 EU/mL at day 29 (1 month after dose one) and continued to increase to 479·70 EU/mL at day 57 (1 month after dose two). By day 390, the GMC decreased to 32·43 EU/mL but remained higher compared with prevaccination (figure 2; appendix p 22). No significant change from baseline was noted in the GMCs of M72-specific antibodies in the placebo group during the trial.

Median magnitude of M72-specific CD4 cells expressing IFN-γ or IL-2 was 0·028% (IQR 0·011–0·093): 28 per 100 000 CD4 cells at baseline, 0·383% (0·177–0·663) at day 57 (1 month after dose two), and 0·266% (0·091–0·464) at day 390. At day 57 and day 390, median magnitudes were notably higher in the vaccine group than in the placebo group (p<0·0001 for each timepoint). Median magnitudes increased from day 1 to day 57 for the M72/AS01_E–4_ group, and values were sustained until the end of the trial (appendix pp 23, 28). The percentage of participants with post-baseline CD4 cell response (based on M72-specific CD4 cells expressing IFN-γ or IL-2) peaked at day 57 and was sustained at day 390 in the M72/AS01_E–4_ group, and this expression was higher than in the placebo group at both days 57 and 390 (table 4; appendix pp 30–31). M72-specific CD8 cells expressing IFN-γ or IL-2 are presented in the appendix (pp 24, 29).

**Table 4:** Cell-mediated immune response: percentage of participants with post-baseline M72-specific CD4 cell response

	**Day 57**	**Day 390**
M72	n=96; 95·8% (90·3–98·7)	n=96; 88·5% (81·0–93·8)
Placebo	n=31; 19·4%* (8·2–36·0)	n=32; 25·0 (12·3–42·0)
p value	p<0·0001	p<0·0001

Data are percentage of participants with positive response (95% CI). Denominators are based on the participants in the per-protocol for cellular immunogenicity population with data available to determine responder status. p value based on Fisher's exact test comparing the percentage of responders between treatment groups. Responder status was based on positive (interferon-γ or interleukin-2) cytokine combination (after baseline) and determined for each participant, T-cell type, treatment group, and visit.

* One sample from the day 57 timepoint was inadvertently not tested.

Reactogenicity and immunogenicity analyses were performed based on IGRA status at baseline. Among participants in the M72/AS01_E–4_ group, those with positive IGRA status at baseline had somewhat higher percentages of solicited adverse events than those with negative IGRA status (appendix p 25), had notably higher GMCs than those with IGRA-negative status at baseline in the M72/AS01_E–4_ group only (appendix p 32) and in both groups (appendix p 33), and had a slightly greater magnitude of M72-specific CD4 cell response than those with IGRA-negative status at baseline (appendix pp 34–35, 38–39). M72-specific CD8 cell responses by IGRA are shown in the appendix (p 37).

## Discussion

In this trial of the M72/AS01_E–4_ vaccine in people living with HIV, a two-dose regimen of M72/AS01_E–4_ vaccine (0·5 mL dose containing 10 μg M72 reconstituted with adjuvant AS01_E–4_), administered 1 month apart, was well tolerated, with an acceptable safety profile, and was immunogenic in antiretroviral-treated participants aged 16–35 years with well controlled HIV.

The M72/AS01_E–4_ vaccine reactogenicity profile, as assessed by solicited adverse events, was consistent with previous M72 trials in people living with or without HIV, including the phase 2b efficacy trial of 3500 adults without HIV.^6^ In the current trial, injection-site pain was the most common adverse event, reported in 83% of vaccine recipients after either dose, followed by headache and fatigue. Most solicited adverse events were mild or moderate in severity and resolved with median duration 3 days or less after vaccination, indicating tolerability of the vaccine.

Unsolicited adverse events related to vaccine were primarily comprised of injection-site reactions reported at day 8 or thereafter. Severe intervention-related unsolicited adverse events were reported in two (1%) participants in the M72/AS01_E–4_ group (the same number as the placebo group). There were no serious adverse events or deaths related to trial vaccine. There were no differences between groups in terms of the percentage of participants with HIV viral loads more than 200 copies per mL or CD4 counts less than 350 cells per μL at any timepoint. The percentage of participants with viral loads of more than 200 copies per mL increased from baseline to the end of the trial visit to a similar extent in both groups, which could be attributed to decreased ART adherence over time.

Humoral and cellular immunogenicity data showed that the M72/AS01_E–4_ vaccine produced robust antibody and CD4 cell responses. These findings are congruent with results from previous M72 trials, irrespective of HIV status, with peak concentrations after dose two and sustained responses to the end of the trial on day 390. The previous M72 trials in people living with and without HIV indicated that immune responses would be sustained through to at least 36 months after vaccination.^7^, ^14^ CD8 T-cell responses were not significantly induced, in line with previous studies of M72/AS01_E–4_ and other adjuvanted protein vaccines, probably because the antigen does not enter the major histocompatibility complex class I presentation pathway.^17^ The importance of CD4 T cells expressing IFN-γ for the control of tuberculosis has been well established, whereas the role of antibody responses has been dismissed for decades but there has now been renewed interest based on promising new data supporting their contribution to tuberculosis control from BCG-vaccinated non-human primates.^18^

In this trial, we enrolled participants with positive and negative IGRA test results at baseline. This approach enabled us to determine that there was a M72/AS01_E–4_ booster effect among participants with IGRA-positive status (who are sensitised by *M tuberculosis*) and a priming effect among participants with IGRA-negative status (who are immunologically naive to *M tuberculosis*). Among M72 vaccine recipients, participants with IGRA-positive status at baseline had GMCs that were higher than those with IGRA-negative status at baseline across all post-baseline timepoints. This difference was notable at 1 month after dose one, and at 6 months and 12 after dose two. The CD4^+^ T-cell response in M72 vaccine recipients with IGRA-positive status was generally slightly higher in M72 vaccine recipients with IGRA-negative status.

Our results support data reported for the M72/AS01_E–4_ trial in people living with HIV in India, where vaccine recipients with baseline IGRA-positive status had higher GMCs than those with baseline IGRA-negative status, during the 30-day interval between the first and second doses.^13^ However, in that trial, no notable differences were seen beyond day 30, which could be attributed to the trial's smaller sample size.^13^ Of note, GMC values among participants with well controlled HIV on ART and IGRA-positive status in our trial (559·5 EU/mL at day 57 and 44·0 EU/mL at day 390) were very similar to those reported for participants without HIV and IGRA-positive status in the M72 phase 2b trial (547·0 EU/mL at month 2 and 41·5 EU/mL at month 12).^7^

Potential associations between IGRA status and reactogenicity were explored: we found that among participants in the M72/AS01_E–4_ group, any injection-site swelling and fever, and severe headache and fatigue appeared to be more frequent among those who were positive for IGRA at baseline. There were no notable differences in the frequencies of solicited adverse events between participants with HIV and baseline IGRA-positive status in our trial compared with participants without HIV and baseline IGRA-positive status in the M72 phase 2b trial.^7^

During the trial, only seven participants reported any signs and symptoms compatible with tuberculosis, and no participants had laboratory-confirmed pulmonary tuberculosis. If we assume an overall tuberculosis incidence of 0·7% (0·7 events per 100 person years)^1^ and 200 person-years accrued in the placebo group, we could have expected to encounter two tuberculosis events in the placebo group during this trial. The absence of tuberculosis events could be due to chance, but it could also be due to the stringent requirements intended to minimise the risk of tuberculosis, including enrolment requirements for stable ART for at least 3 months, previous completion of tuberculosis preventive therapy, favourable HIV viral load and CD4 cell counts, exclusion of past tuberculosis, and a negative sputum for tuberculosis at baseline.

The main strength of this trial is its inclusion of a larger population of people living with HIV in a tuberculosis-endemic country, compared with the previously completed studies.^12^, ^13^, ^14^ This trial provided additional data on people living with HIV, contributing to the decision to include people living with HIV in the M72 phase 3 trial. Another strength of this trial is the opportunity to evaluate the effect of reactogenicity and immunogenicity of M72 versus placebo, among participants with baseline IGRA positivity and negativity.

One of the limitations of our trial is that the trial population was restricted to people living with HIV who were virally suppressed on ART. Therefore, our results might not represent the general population of people living with HIV, including those not taking ART, not taking ART as recommended, or with newly diagnosed HIV. Another limitation was that 88% of the participants were female, considering that the risk of tuberculosis is higher in males.^1^, ^19^ This pattern has also been observed in trials of people living with HIV in similar settings^20^, ^21^ and is probably due to a number of factors, including female individuals being more likely to interact with the health-care system, to volunteer for trial participation, and to test for HIV,^22^ and female individuals in South Africa having higher HIV prevalence than male individuals.^23^ Although possible that disproportionate enrolment of females in this trial could impact overall trial outcomes, this is unlikely given that safety and immunogenicity outcomes were consistent with previous trials throughout the M72 programme, irrespective of HIV status.

Another potential limitation is attrition bias due to missing outcome data. Overall, 26 (7%) participants discontinued early: seven (4%) in the M72/AS01_E–4_ group and 19 (10%) in the placebo. Although the discontinuation rate differs, there were no notable differences between the groups with respect to demographic or baseline characteristics when comparing safety and per-protocol populations. Furthermore, the higher number of early discontinuations in the placebo group is unlikely to affect the trial objectives.

In conclusion, a two-dose regimen of M72/AS01_E–4_ vaccine, administered 1 month apart, was well tolerated, with an acceptable safety profile, and was immunogenic in virally suppressed, ART-treated people living with HIV aged 16–35 years. The timely completion of this trial supported the decision to include a larger number of people living with HIV in the M72/AS01_E–4_ global registration phase 3 trial.

For the **protocol** see https://cdn.clinicaltrials.gov/large-docs/81/NCT04556981/Prot_000.pdf

### Contributors

### Data sharing

Anonymised participant-level data can be shared with external researchers in accordance with the trial participants' written and executed informed consent document and any local or applicable regulations on data sharing. Qualified researchers can submit a request for anonymised participant-level data along with a research proposal to Gates MRI for review. The types of supporting information that could be shared with external researchers include: the study protocol, statistical analysis plan, informed consent form, clinical study report, and analytic code. A data sharing agreement must be in place before any clinical trial data are shared. There are additional circumstances that could prevent the sharing of data with external researchers, including but not limited to contractual obligations to existing partners and any restrictions imposed by regulatory bodies.

## Declaration of interests

At the time this trial was designed, initiated, and conducted, AFD, ACS, LLH, MD, LS, AC, JA, DB, and NF were employees of the Gates Medical Research Institute, and declare no competing interests. RJW receives funding from Wellcome (grant number 226817). He is supported by the Francis Crick Institute, which receives funding from Wellcome (grant number CC2112), UK Research and Innovation (grant number CC2112), and Cancer Research UK (grant number CC2112). He also receives support in part from the National Institute for Health and Care Research Biomedical Research Center of Imperial College National Health Service Trust and received support from the Wellcome Trust and the Gates Foundation to travel to investigator meetings. KM receives funding from the Gates Medical Research Institute (grant number Gates MRI–TBV02–301-DTHF), TB Alliance (grant number NC-009-DTHF), and the National Institutes of Health (grant number UCT00041179, DTHC). She also received travel support from and provided unpaid leadership at the International Union Against Tuberculosis and Lung Disease. LF, JCI, KN, and MT declare no competing interests.
