# Supplementary material for: Safety and immunogenicity of investigational tuberculosis vaccine M72/AS01E–4 in people living with HIV in South Africa: an observer-blinded, randomised, controlled, phase 2 trial
Source: Lancet HIV. 2025 Jul 1;12(8):e546–55. doi: 10.1016/S2352-3018(25)00124-9 (PMC12310912; doi:10.1016/S2352-3018(25)00124-9)
Supplement: Supplementary appendix [file mmc1.pdf]

THE LANCET
HIV

Supplementary appendix

This appendix formed part of the original submission and has been peer reviewed. We post it as supplied by the authors.

Supplement to: Dagnew AF, Han LL, Naidoo K, et al. Safety and immunogenicity of investigational tuberculosis vaccine M72/AS01$_{E-4}$ in people living with HIV in South Africa: an observer-blinded, randomised, controlled, phase 2 trial. *Lancet HIV* 2025; published online July 1. https://doi.org/10.1016/S2352-3018(25)00124-9.

Supplementary Materials

Table of Contents

METHODS- TRIAL DESIGN, PARTICIPANTS, AND ANALYSES 4
IMMUNOGENICITY METHODS 7
PREGNANCY 26
Supplementary Table 1 Summary of Laboratory Assays 5
Supplementary Table 2 T cell ICS Flow Panel Markers and Fluorochromes 6
Supplementary Table 3 Determination of M72-Specific Response by Composite Measure of Baseline and Post-Baseline M72-Specific T Cell Responder Status 10
Supplementary Table 4 Summary of Demographic Data and Key Baseline Characteristics in the Per Protocol Population 11
Supplementary Table 5 Duration of Injection Area Symptoms and Any General Body Symptoms Within 7 Days after Each Dose as Reported on the Diary Card (Safety Population) 12
Supplementary Table 6 Unsolicited Adverse Events by Decreasing Frequency of Participants with Preferred Term (Safety Population) 14
Supplementary Table 7 Related Unsolicited Adverse Events by Decreasing Frequency of Participants with Preferred Term Safety Population 16
Supplementary Table 8 Summary of Hematology Parameters 17
Supplementary Table 9 Summary of Serum Chemistry Parameters 18
Supplementary Table 10 Summary of HIV Viral Load (copies per mL) per Visit 19
Supplementary Table 11 Number and Percentage of Participants with $CD4^{+}$ T-Cell Counts <350 cells per µL Safety Population) 20
Supplementary Table 12 Seropositivity Measured by ELISA by Timepoint (Per Protocol Population) 21
Supplementary Table 13 M72/AS01$_{E-4}$ Geometric Mean Antibody Concentration Results for the M72/AS01$_{E-4}$ Vaccine Group by Timepoint (Per Protocol Population) 22
Supplementary Table 14 Magnitude of M72 $CD4^{+}$T Cell Positive IFN-γ and/or IL-2) Cytokine Response by Visit and Change from Baseline Day 1) Per Protocol for Cellular Immunogenicity Population 23
Supplementary Table 15 Magnitude of M72 $CD8^{+}$ T Cell Positive IFN-γ and/or IL-2) Cytokine Response by Visit and Change from Baseline Day 1) Per Protocol for Cellular Immunogenicity Population 24
Supplementary Table 16 Percentages of Participants with Solicited AEs Based on IGRA Status at Baseline (Safety Population) 25
Supplementary Table 17 Geometric Mean Antibody Concentrations by IGRA for Participants in the M72/AS01$_{E-4}$ Group (Per Protocol for Cellular Immunogenicity Population) 32
Supplementary Table 18 Cell-Mediated Immune Response: Percentage of Participants with Post-Baseline M72-Specific CD4+ T-Cell Response by IGRA Status at Baseline (Per Protocol for Cellular Immunogenicity Population) 36

Supplementary Table 19 Magnitude of M72 CD4$^+$T Cell Positive IFN-γ and/or IL-2 Cytokine Response by Visit and Change from Baseline Day 1 among IGRA Positive Participants at Baseline (Per Protocol for Cellular Immunogenicity Population) .38
Supplementary Table 20 Magnitude of M72 CD4$^+$T Cell Positive IFN-γ and/or IL-2 Cytokine Response by Visit and Change from Baseline Day 1 among IGRA Negative Participants at Baseline (Per Protocol for Cellular Immunogenicity Population) .39

Supplementary Figure 1 Gating Strategy for the T Cell ICS Flow Cytometry Panel........... 8
Supplementary Figure 2 Percentage of Participants Reporting Solicited Adverse Events 27
Supplementary Figure 3 Longitudinal Magnitude of CD4$^+$ T-cell Cytokine IFN-γ and/or IL-2 Response by Participant (Per Protocol for Cellular Immunogenicity Population).. 28
Supplementary Figure 4 Longitudinal Magnitude of CD8$^+$ T-cell Cytokine IFN-γ and/or IL-2 Response by Participant (Per Protocol for Cellular Immunogenicity Population).. 29
Supplementary Figure 5 M72-Specific CD4$^+$ T-cell Polyfunctional Plots (Per Protocol for Cellular Immunogenicity Population)... 30
Supplementary Figure 6 Geometric Mean Antibody Concentrations Overall and by IGRA Status at Baseline (Per Protocol Population) 33
Supplementary Figure 7 Magnitude of M72-Specific CD4$^+$ T-cell IFN-γ and/or IL-2 Response, by IGRA Status at Baseline, M72/AS01$_{E-4}$ Group Only (Per Protocol for Cellular Immunogenicity Population) 34
Supplementary Figure 8 Longitudinal Magnitude of M72-Specific CD4$^+$ T-cell IFN-γ and/or IL-2 Response by IGRA Status at Baseline (Per Protocol for Cellular Immunogenicity Population)... 35
Supplementary Figure 9 Longitudinal Magnitude of CD8$^+$ T-Cell IFN-γ and/or IL-2 Response by IGRA status at Baseline (Per Protocol for Cellular Immunogenicity Population) 37

METHODS- TRIAL DESIGN, PARTICIPANTS, AND ANALYSES

A total of 401 participants 16 to 35 years of age received at least one dose of M72/AS01$_{E-4}$ or placebo and were enrolled at six clinical trial sites in South Africa. These sites included the South African Tuberculosis Vaccine Initiative (SATVI), the Center for the AIDS Program of the Research in South African eThekweni Clinical Research Site, Shandukani Wits RHI, the Aurum Institute-Clinical Research Initiative, the Desmond Tutu HIV Foundation, and the Center for infectious Diseases Research in Africa (CIDRI) Africa University of Cape Town.

The Ethics Committees that reviewed and approved the study were the University of Capetown Human Research Ethics Committee (HREC), registration number REC-210208-007, the Biomedical Research Ethics Committee, Univ KwaZulu Natal registration number REC-290408-009, and the University of Witwatersrand, registration number REC-250208-004.

Exclusion criteria included current or previous TB, clinically significant medical conditions other than HIV infection, medications or other therapies that may have impacted the immune system within 90 days prior to Day 1, receipt or donation of blood or blood products within 90 days prior to Day 1, receipt of any vaccine in the period starting seven days before and ending seven days after each dose, abnormal laboratory values, and females who were pregnant or lactating or who were not willing to avoid pregnancy through one year after the first dose.

Supplementary Table 1 Summary of Laboratory Assays

Test	Sample Type	Manufacturer	Instrument	ASSAY
Hemoglobin, white blood cells, platelets	Whole Blood	ROCHE (USA)	Sysmex XN1000	Roche/Sysmex - Cell pack DCL, Fluorocell WNR
Chemistry	Serum	ROCHE (USA)	COBAS INTEGRA 400	Roche Integra
Urinalysis	Urine	ROCHE (USA)	Roche Combur 10 (Manual Method)	Roche Combur 10 (Manual Method)
QFT IGRA	Plasma	DYNEX (USA)	DS2	QuantiFERON-TB Gold Plus (QFT-Plus)
GeneXpert	Sputum	Cepheid (USA)	GeneXpert 4	MTB RIF ULTRA
CD4	Whole Blood	Becton Dickinson (USA)	FacsCanto II	Mutlitest Trucount Flowcytometry
BHCG	Serum	ABBOTT (USA)	Alinity I	Chemiluminescent microparticle immunoassay ELISA
HIV ELISA	Serum	ABBOTT (USA)	Alinity I	Chemiluminescent microparticle immunoassay ELISA
HIV viral load	Plasma	ABBOTT (USA)	ABBOTT M2000RT	Realtime HIV-1
HEP Serology (hepatitis B and C)	Serum	ABBOTT (USA)	Alinity I	Chemiluminescent microparticle immunoassay ELISA

All assays were conducted by Bio Analytical Research Corporation, South Africa
IGRA sputum samples were collected for GeneXpert test for *M. tuberculosis* on a polymerase-chain-reaction (PCR) assay, for eligibility and for signs and/or symptoms of TB.
Hematology and serum chemistry tests were performed on Days 1 and 29, and HIV viral load and $CD4^{+}$ T-cell counts were performed on Days 1, 57, 210, and 390. Blood samples for immunogenicity were collected on Day 1 and Day 29 (prior to dose administration), and on Days 57, 210, and 390.

Supplementary Table 2 T cell ICS Flow Panel Markers and Fluorochromes

Laser	Fluorochrome	Marker	Intracellular (I) or Extracellular (E) Staining	Vendor	Catalog #	Clone	Vol / 100µL test (µL)
Violet 407 nm	BV421	CD40L	I	BioLegend (USA)	310824	24-31	1·25
	Zombie Aqua	Viability	N/A	BioLegend (USA)	423102	N/A	0·50
	BV605	CD8	E	BioLegend (USA)	301040	RPA-T8	2·50
Blue 488nm	Alexa Fluor 488	TNF	I	BioLegend (USA)	502915	MAb11	5·00
	PerCP-Cy5·5	CD4	I	BioLegend (USA)	300530	RPA-T4	2·50
Yellow 561nm	PE	IL-2	I	BioLegend (USA)	500307	MQ1-17H12	2·50
Red 633nm	APC	IFNg	I	BioLegend (USA)	506510	B27	5·00
	Alexa Fluor 700	CD3	E	BioLegend (USA)	300424	UCHT1	2·50

IMMUNOGENICITY METHODS

Flow ICS method

Procedure: Cryopreserved PBMCs were thawed in RPMI complete medium with DNase I (STEMCELL™ Technologies, Canada) and counted (Nexcelom Cellometer Auto 2000 Cell Counter with ViaStain AOPI Staining Solution, USA) prior to resting overnight in complete medium [RPMI without phenol red (GIBCO, United Kingdom), 10% fetal bovine serum (GIBCO, USA), 50 µg/mL gentamicin (Sigma-Aldrich, Israel), 2mM L-glutamine (GIBCO, USA)] at 37°C and 5% CO_2. The next morning, the cells were counted and plated at one million PBMC per well. Wells were treated with either of the following conditions for 6 hours at 37°C and 5% CO_2.

1. Staphylococcal enterotoxin b (SEB, Toxin Technology Inc, USA): 10 µg/mL of SEB in the presence of 1 µg/mL of anti-CD28 (BD Biosciences, USA)/CD49d (BD Biosciences) co-stimulatory molecules
2. M72 peptide pool (178 peptides covering the M72 protein with 11 aa overlaps)(B2S Life Sciences, USA): 1 µg/mL of M72 peptide pool in the presence of 1 µg/mL of anti-CD28/CD49d co-stimulatory molecules
3. Dimethyl sulfoxide (DMSO; Sigma, United Kingdom) Unstimulated Control: 0.1% of DMSO in the presence of 1 µg/mL of anti-CD28/CD49d co-stimulatory molecules
4. HIV GAG peptide pool (JPT Peptide Technologies, PepMix™ HIV-1 GAG Ultra, USA): 1 µg/mL of HIV GAG peptide pool in the presence of 1 µg/mL of anti-CD28/CD49d co-stimulatory molecules

After the initial 2hrs of stimulation, 10 µL of the Brefeldin A (GolgiPlug, BD Biosciences, USA)/Monensin (GolgiStop, BD Biosciences, USA) mixture (1X final) was added to all wells before continuing incubation for an additional 4 hours at 37°C and 5% CO2. Cells were then centrifuged and washed in 1X Dulbecco's phosphate buffered saline without calcium or magnesium (DPBS; Corning, USA) and incubated with Zombie Aqua cell viability dye for 15 minutes at room temperature. The cells were then washed twice with staining buffer (SB) containing DPBS, 1% Bovine Serum Albumin (BSA; Miltenyi Biotec, Germany), 10mM HEPES (GIBCO, United Kingdom), 1mM EDTA (Invitrogen, USA), and 0.01% Sodium Azide (VWR, USA) prior to blocking the Fc receptors with Human TruStain FcX (BioLegend, USA) for 15 minutes at 4°C. After two washes with SB, the cells were stained for 30 minutes at 4°C with surface antibodies, washed twice with SB, fixed with 1X BD CytoFix buffer (BD Biosciences, USA) for 30 minutes at 4°C, and washed twice with 1X Perm/Wash Buffer (BD Cytofix/Cytoperm/Fixation/Permeabilization Kit, BD Biosciences, USA; Water, Quality Biological, USA) prior to intracellular staining for 30 minutes at 4°C. After incubation, the cells were washed twice with 1X Perm/Wash Buffer and then resuspended in a final volume of 400 µL SB.

Equipment and software: All samples were acquired on a single BD FACSCanto flow cytometer to minimize variability. Flow cytometer performance was monitored and maintained using BD FACSDiva CS&T Beads (BD Biosciences, USA). Data analysis was performed using FlowJo software (version 10.8). BD FACSDiva version 9.0 Application Settings were created to ensure consistency throughout the course of the study.

Supplementary Figure 1 Gating Strategy for the T Cell ICS Flow Cytometry Panel

a. Gating Strategy for the T-Cell ICS Flow Cytometry Panel and 1b sample

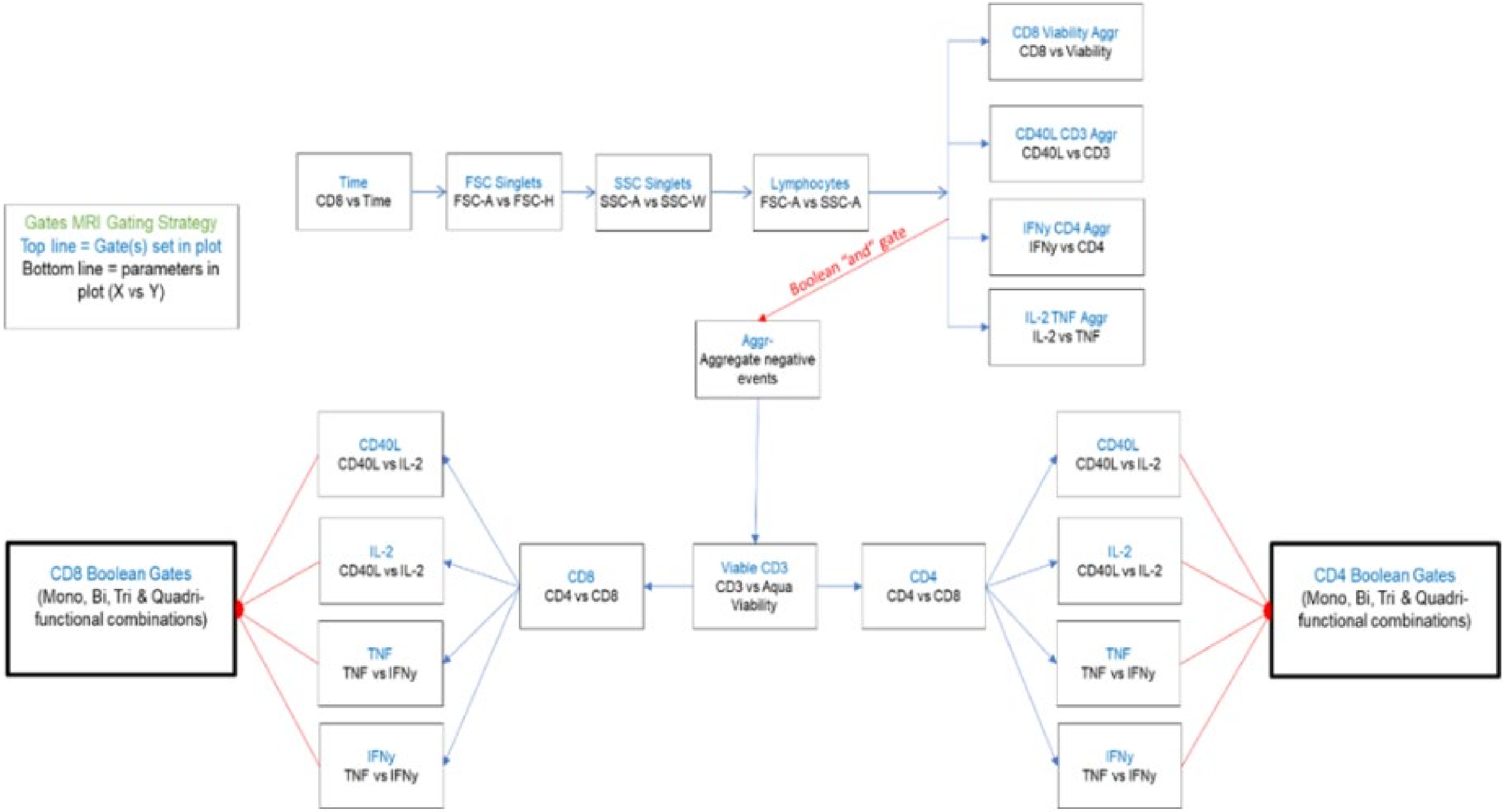

b. Representative plots for ICS Gating Strategy noted in F for the T Cell ICS Flow Cytometry Panel (example from SEB- -stimulated sample

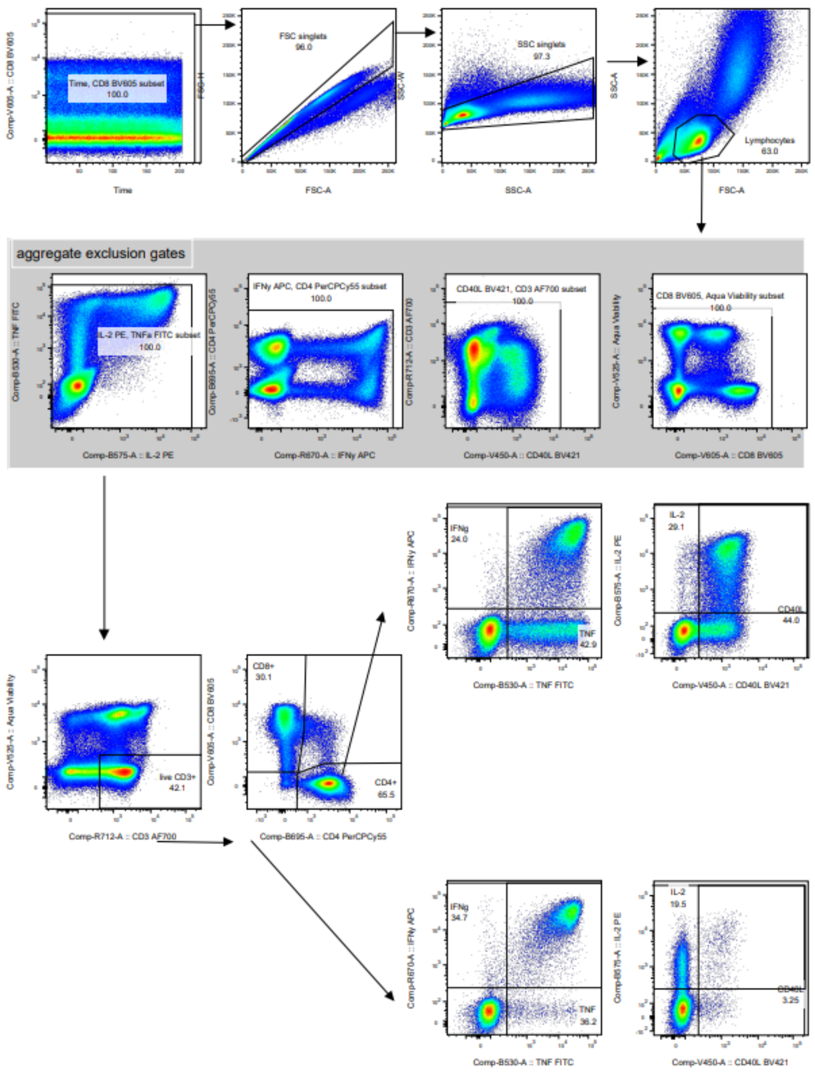

Supplementary Table 3 Determination of M72-Specific Response by Composite Measure of Baseline and Post-Baseline M72-Specific T Cell Responder Status

Baseline M72-specific T cell	Post-baseline M72-specific T cell	M72-specific response		
Negative	Negative	Negative		
Negative	Positive	Positive		
Positive	Negative	Negative		
Positive	Positive	Determine status with odds ratio and Breslow-Day test:		
		Breslow-Day test p-value	Difference in log odds ratio	Responder Status
		≥0·05	<0	Negative
		≥0·05	>0	Negative
		<0·05	<0	Negative
		<0·05	>0	Positive

The Breslow-Day test was utilized as a tool to determine the responder status. If the baseline and post-baselines ORs differ significantly based on the Breslow-Day test, and the difference in the log OR indicates post baseline is greater than baseline, the responder status is positive.

Supplementary Table 4 Summary of Demographic Data and Key Baseline Characteristics in the Per Protocol Population

Characteristic	M72/AS01$_{E-4}$ (N=143)	Placebo (N=130)
Age (years) mean (SD)	29·3 (4·36)	30·2 (3·88)
Sex assigned at birth n (%)		
Male	21 (15)	16 (12)
Female	122 (85)	114 (88)
Race n (%)		
Black	138 (97)	127 (98)
South African Colored	5 (3)	3 (2)
IGRA-positive at baseline n (%)	63 (44)	62 (48)
IGRA-negative at baseline n (%)	80 (56)	68 (52)
Weight (kg) mean (SD)	73·1 (19·73)	75·9 (19·62)
Height (cm) mean (SD)	161·8 (7·39)	161·5 (7·98)
BMI (kg/m^2) mean (SD)	27·9 (7·19)	29·2 (7·71)
CD4$^+$ T-cell count -cells per µL 200-349 n (%)	4 (3)	2 (2)
CD4$^+$ T-cell count - cells per µL 350-499 n (%)	15 (10)	15 (12)
CD4$^+$ T-cell count - cells per µL ≥ 500 n (%)	124 (87)	113 (87)
HIV viral load (copies per mL) Not Detected* n (%)	115 (80)	105 (81)
HIV viral load (copies per mL) ≤ 200 n (%)	26 (18)	23 (18)
HIV viral load (copies per mL) > 200 n (%)	2 (1)	2 (2)

Percentages calculated using the number of participants in each trial group in the relevant analysis set, with data available, as the denominator

N, number of participants in the Safety Population; SD, standard deviation; BMI, body mass index (calculated using raw data measurements); CD4$^+$, cluster of differentiation 4

HIV = human immunodeficiency virus; IGRA = interferon-γ release assay

South African Colored refers to people of mixed race.

*Samples for which HIV viral load was detectable but not quantifiable

Supplementary Table 5 Duration of Injection Area Symptoms and Any General Body Symptoms Within 7 Days after Each Dose as Reported on the Diary Card (Safety Population)

	Dose 1		Dose 2	
	$M72/AS01_E$ (N = 201)	Placebo (N = 200)	$M72/AS01_E$ (N = 201)	Placebo (N = 200)
**Injection Area Symptoms**				
**Pain (Any) n/N1 (%)**	139/201 (69)	40/195 (21)	140/183 (77)	26/184 (14)
Mean (SD)	2·9 (1·99)	1·6 (1·30)	2·8 (1·5)	1·7 (0·96)
Mean Days (SD)	2·9 (1·99)	1·6 (1·30)	2·8 (1·5)	1·7 (0·96)
Median Days	2	1	2	2
Q1, Q3	1, 4	1, 2	2, 4	1, 2
Min, Max	1, 7	1, 7	1, 7	1, 5
**Redness (Any) n/N1 (%)**	46/198 (23)	17/196 (9)	45/178 (25)	7/186 (4)
Mean Days (SD)	2·8 (2·13)	1·8 (1·20)	3·3 (2·09)	2·0 (1·91)
Median Days	2	1	3	1
Q1, Q3	1, 4	1, 2	2, 5	1, 3
Min, Max	1, 7	1, 5	1, 7	1, 6
**Swelling (Any) n/N1 (%)**	62/197 (32)	11/196 (6)	61/181 (34)	8/187 (4)
Mean Days (SD)	3.5 (2.05)	2·2 (1.54)	3·3 (1·95)	1·9 (1·13)
Median Days	3	2	3	2
Q1, Q3	2, 5	1, 3	2, 4	1, 3
Min, Max	1, 7	1, 6	1, 7	1, 4
**General Body Symptoms**				
**Fever (Any) n/N1 (%)**	39/200 (20)	21/195 (11)	62/184 (34)	31/185 (17)
Mean Days (SD)	1·6 (1·07)	1·3 (0·78)	1·7 (1·11)	1·6 (0·91)
Median Days	1	1	1	1
Q1, Q3	1, 2	1, 1	1, 2	1, 2
Min, Max	1, 5	1, 4	1, 7	1, 4
**Headache (Any) n/N1 (%)**	92/200 (46)	61/196 (31)	104/183 (57)	44/188 (23)
Mean Days (SD)	2·2 (1·53)	2·4 (1·80)	2·7 (1·70)	2·3 (1·62)
Median Days	2	2	2	2
Q1, Q3	1, 3	1, 3	1, 4	1, 3
Min, Max	1, 7	1, 7	1, 7	1, 7
**Fatigue (Any) n/N1 (%)**	81/199 (41)	64/196 (33)	95/183 (52)	41/187 (22)
Mean Days (SD)	2·3 (1·66)	2·1 (1·42)	2·7 (1·82)	2·1 (1·48)
Median Days	2	2	2	1
Q1, Q3	1, 3	1, 3	1, 3	1, 3
Min, Max	1, 7	1, 7	1, 7	1, 6
**GI Symptoms (Any) n/N1 (%)**	33/199 (17)	26/196 (13)	32/183 (18)	16/187 (9)
Mean Days (SD)	2·2 (1·32)	2.5 (1·65)	2·8 (2·14)	1·9 (1·12)
Median Days	2	2	2	2
Q1, Q3	1, 3	1, 3	1, 3	1, 3
Min, Max	1, 5	1, 6	1, 7	1, 5
**Myalgia (Any) n/N1 (%)**	58/199 (29)	35/196 (18)	73/183 (40)	22/188 (12)
Mean Days (SD)	2·8 (1·84)	2 (1·37)	2·9 (1·73)	2·7 (1·67)
Median Days	2	2	2	2

Q1, Q3	2, 3	1, 2	2, 3	1, 3
Min, Max	1, 7	1, 7	1, 7	1, 7

Data reported as collected on the diary card from Days 1 to 7 after dose

Participants were categorized under the highest severity experienced during Days 1 to 7 after dose·

SD, Standard deviation; Q1, Quartile 1; Q3, Quartile 3; Max, maximum; Min, minimum; N, number of participants who received a dose; N1, number of participants with diary results; n, number of participants reporting any AE; GI, gastrointestinal

Supplementary Table 6 Unsolicited Adverse Events by Decreasing Frequency of Participants with Preferred Term (Safety Population)

Preferred Term	M72/AS01$_{E-4}$ N=201			Placebo N=200		
	n	%	Events	n	%	Events
Headache	18	9	24	25	13	34
Cough	10	5	10	6	3	7
Dizziness	8	4	8	9	5	9
Injection site erythema	7	4	7	1	1	1
Myalgia	7	4	7	0	0	0
Upper respiratory tract infection	7	4	7	5	3	5
Back pain	6	3	8	4	2	4
Influenza like illness	6	3	6	3	2	3
Diarrhea	5	3	5	2	1	2
Injection site pruritus	5	3	5	1	1	1
Alanine aminotransferase increased	4	2	4	5	3	6
Conjunctivitis allergic	4	2	4	0	0	0
Influenza	4	2	5	6	3	6
Rash	4	2	4	2	1	2
Abdominal pain upper	3	2	3	0	0	0
Arthralgia	3	2	3	1	1	2
Aspartate aminotransferase increased	3	2	3	6	3	6
Blood pressure increased	3	2	3	1	1	1
Haemoglobin decreased	3	2	3	2	1	2
Injection site swelling	3	2	3	0	0	0
Nasal congestion	3	2	3	0	0	0
Pyrexia	3	2	3	4	2	4
Tonsillitis	3	2	3	0	0	0
Toothache	3	2	3	0	0	0
Dysfunctional uterine bleeding	2	1	3	4	2	4
Feeling cold	2	1	2	0	0	0
Hypertension	2	1	2	0	0	0
Injection site bruising	2	1	2	0	0	0
Injection site induration	2	1	2	0	0	0
Injection site pain	2	1	2	0	0	0
Iron deficiency anemia	2	1	2	1	1	1
Metrorrhagia	2	1	2	1	1	1
Muscle spasms	2	1	2	0	0	0
Musculoskeletal chest pain	2	1	2	0	0	0
Pain in extremity	2	1	2	2	1	2
Rhinitis	2	1	2	2	1	2
Sinusitis	2	1	2	0	0	0
Urinary tract infection	2	1	2	4	2	4
Vaginal discharge	2	1	2	0	0	0
Vomiting	2	1	2	2	1	2
Abdominal pain lower	1	1	1	0	0	0
Anaemia	1	1	1	0	0	0
Axillary mass	1	1	1	0	0	0
Blood pressure abnormal	1	1	1	0	0	0
Blood pressure diastolic increased	1	1	1	0	0	0
Body tinea	1	1	1	0	0	0
Bone swelling	1	1	1	0	0	0
COVID-19	1	1	1	0	0	0
Cellulitis	1	1	1	0	0	0
Chest pain	1	1	1	2	1	2
Conjunctival hyperemia	1	1	1	0	0	0
Constipation	1	1	1	1	1	2
Decreased appetite	1	1	1	0	0	0
Dry skin	1	1	1	0	0	0
Dyspepsia	1	1	1	0	0	0
Ear pain	1	1	1	0	0	0
Essential hypertension	1	1	1	0	0	0
Fatigue	1	1	2	1	1	1
Furuncle	1	1	1	1	1	1
Gastro-esophageal reflux disease	1	1	1	1	1	1
Hemorrhoids	1	1	1	0	0	0
Hypersensitivity	1	1	1	1	1	1
Increased appetite	1	1	1	0	0	0

Preferred Term	M72/AS01$_{E-4}$ N=201			Placebo N=200		
	n	%	Events	n	%	Events
Injection site cellulitis	1	1	1	0	0	0
Lower respiratory tract infection	1	1	1	0	0	0
Lymphadenitis	1	1	1	0	0	0
Nausea	1	1	1	2	1	2
Oropharyngeal pain	1	1	1	2	1	2
Pelvic pain	1	1	1	1	1	1
Periorbital swelling	1	1	1	0	0	0
Platelet count decreased	1	1	1	0	0	0
Pruritus	1	1	1	2	1	2
Pustule	1	1	1	0	0	0
Pyelonephritis	1	1	1	0	0	0
Rash pruritic	1	1	1	0	0	0
Subcutaneous abscess	1	1	1	0	0	0
Swelling	1	1	1	0	0	0
Thermal burn	1	1	1	1	1	1
Throat irritation	1	1	1	0	0	0
Tibia fracture	1	1	1	0	0	0
Vaginal hemorrhage	1	1	1	0	0	0
Vulvovaginal candidiasis	1	1	1	0	0	0
Weight decreased	1	1	1	0	0	0
White blood cell count decreased	1	1	1	0	0	0
Abdominal pain	0	0	0	1	1	1
Abortion spontaneous	0	0	0	1	1	1
Breast inflammation	0	0	0	1	1	1
Carbuncle	0	0	0	1	1	1
Conjunctivitis	0	0	0	2	1	3
Dry eye	0	0	0	1	1	1
Dysmenorrhea	0	0	0	1	1	1
Epilepsy	0	0	0	1	1	1
Epistaxis	0	0	0	1	1	1
Escherichia urinary tract infection	0	0	0	1	1	1
Eye pruritus	0	0	0	1	1	1
Eye swelling	0	0	0	1	1	1
Flank pain	0	0	0	3	2	3
Hepatitis alcoholic	0	0	0	1	1	1
Insomnia	0	0	0	1	1	1
Iron deficiency	0	0	0	1	1	1
Limb injury	0	0	0	1	1	1
Menorrhagia	0	0	0	5	3	5
Night sweats	0	0	0	1	1	1
Pain in jaw	0	0	0	1	1	1
Physical assault	0	0	0	1	1	1
Restlessness	0	0	0	1	1	1
Rhinorrhea	0	0	0	1	1	1
Sinus pain	0	0	0	1	1	1
Sneezing	0	0	0	1	1	1
Suspected COVID-19	0	0	0	1	1	1
Tension headache	0	0	0	1	1	1
Transaminases increased	0	0	0	1	1	1
Tremor	0	0	0	1	1	1
Urinary tract infection staphylococcal	0	0	0	1	1	1
Vulvovaginal discomfort	0	0	0	1	1	1
Vulvovaginal rash	0	0	0	1	1	1

AE, Adverse event; N, number of participants in the Safety Population; Participants with multiple AEs within a preferred term (PT) were counted only once. MedDRA version 23.1 was used for coding and reporting AEs. Decreasing frequency based on M72AS01$_{E-4}$ group

Includes AEs with onset on or after each dose and through the post dose reporting window (28 days after each dose).

Supplementary Table 7 Related Unsolicited Adverse Events by Decreasing Frequency of Participants with Preferred Term Safety Population

Preferred Term	M72/AS01$_{E-4}$ N=201			Placebo N=200		
	n	%	Events	n	%	Events
Number of Participants with at least 1 Related Unsolicited AE	25	12	35	11	6	15
Injection site erythema	7	4	7	1	1	1
Dizziness	6	3	6	5	3	5
Injection site pruritus	5	3	5	1	1	1
Injection site swelling	3	2	3	0	0	0
Injection site bruising	2	1	2	0	0	0
Injection site induration	2	1	2	0	0	0
Injection site pain	2	1	2	0	0	0
Abdominal pain upper	1	1	1	0	0	0
Back pain	1	1	1	0	0	0
Cellulitis	1	1	1	0	0	0
Headache	1	1	1	0	0	0
Hypersensitivity	1	1	1	1	1	1
Injection site cellulitis	1	1	1	0	0	0
Nausea	1	1	1	0	0	0
Vomiting	1	1	1	1	1	1
Alanine aminotransferase increased	0	0	0	1	1	1
Aspartate aminotransferase increased	0	0	0	2	1	2
Fatigue	0	0	0	1	1	1
Oropharyngeal pain	0	0	0	1	1	1
Pruritus	0	0	0	1	1	1

AE, Adverse event; N, number of participants in the Safety population; n, number of participants with specified AE
Participants with multiple adverse events within a PT were counted only once.
MedDRA version 23·1 was used for coding and reporting adverse events.
Includes AEs with onset on or after dose 2 and through the post dose reporting window (28 days).

All unsolicited AEs related to trial intervention resolved within 10 days, except for four AEs in the M72/AS01$_{E-4}$ group which resolved within 15 to 24 days (injection site erythema, injection site itchiness, and back pain), and one mild AE of elevated AST in the placebo group which spanned the entire trial.

Supplementary Table 8 Summary of Hematology Parameters

Lab Parameter	M72/AS01$_{E-4}$ N=201 Values SI unit	M72/AS01$_{E-4}$ N=201 Change from Baseline	Placebo N=200 Values SI unit	Placebo N=200 Change from Baseline
**Hemoglobin (g/L)**				
Baseline				
n	201		200	
Mean (SD)	132 (14·75)		133·2 (14·26)	
Median	132		134	
IQR (Q1, Q3)	(123, 140)		(124, 142)	
Min, Max	90, 177		93, 174	
Visit 3, Day 29				
n	197	197	191	191
Mean (SD)	130·1 (14·43)	-1·8 (7·80)	131·9 (14·60)	-1·1 (7·55)
Median	130	-1	133	-1
IQR (Q1, Q3)	(121, 139)	(-7, 3)	(123, 142)	(-6, 3)
Min, Max	97, 174	-24, 17	95, 170	-26, 23
**Leukocytes (10^9/L)**				
Baseline				
n	201		200	
Mean (SD)	5·906 (1·75)		6·116 (1·98)	
Median	5·86		6·01	
IQR (Q1, Q3)	(4.69, 6.96)		(4.52, 7.29)	
Min, Max	1·84, 11·38		2·51, 13·75	
Visit 3, Day 29				
n	197	197	191	191
Mean (SD)	5·981 (1·74)	0.058 (1·27)	6·051 (1·97)	-0·102 (1.52)
Median	5·87	-0·03	5·90	0
IQR (Q1, Q3)	(4.80, 7.10)	(-0.60, 0.69)	(4.64, 7.22)	(-0.83, 0.55)
Min, Max	2·2, 12·34	-4·38, 5·5	2·7, 16·47	-5·41, 6·88
**Platelets (10^9/L)**				
Baseline				
n	201		200	
Mean (SD)	318·1 (86·82)		321·4 (82·31)	
Median	313		313	
IQR (Q1, Q3)	(265, 355)		(265, 371)	
Min, Max	130, 740		141, 624	
Visit 3, Day 29				
n	197	197	191	191
Mean (SD)	312·2 (82·19)	-7·1 (49·79)	312·8 (75·36)	-8·8 (50·33)
Median	307	-7	301	-9
IQR (Q1, Q3)	(257, 356)	(-29, 16)	(267, 358)	(-34, 16)
Min, Max	109, 688	-272, 179	137, 584	-211, 159

SD, Standard Deviation; IQR, Interquartile range; Baseline defined as last non-missing assessment prior to first dose.

Supplementary Table 9 Summary of Serum Chemistry Parameters

Lab Parameter	M72/AS01$_{E-4}$ N=201 Values SI unit	M72/AS01$_{E-4}$ N=201 Change from Baseline	Placebo N=200 Values SI unit	Placebo N=200 Change from Baseline
**Alanine Aminotransferase (U/L)**				
Baseline				
n	201		200	
Mean (SD)	20·9 (11·87)		22·4 (16·91)	
Median	18		17	
IQR (Q1, Q3)	(13, 25)		(13, 25)	
Min, Max	5, 98		8, 144	
Visit 3, Day 29				
n	197	197	191	191
Mean (SD)	20·8 (14·14)	-0·1 (12·65)	22·7 (20.54)	0·4 (21·29)
Median	17	-1	17	-1
IQR (Q1, Q3)	(13, 23)	(-4, 3)	(13, 26)	(-5, 3)
Min, Max	4, 105	-56, 61	6, 214	-114, 200
**Aspartate Aminotransferase (U/L)**				
Baseline				
n	201		200	
Mean (SD)	23·6 (9·24)		25·4 (17·09)	
Median	22		22	
IQR (Q1, Q3)	(18, 25)		(18, 28)	
Min, Max	12, 78		11, 224	
Visit 3, Day 29				
n	197	197	191	191
Mean (SD)	23·1 (9·83)	-0.5 (8·92)	26·0 (21.05)	0·6 (25·41)
Median	21	-1	22	0
IQR (Q1, Q3)	(17, 25)	(-4, 2)	(18, 28)	(-3, 2)
Min, Max	12, 77	-32, 47	8, 232	-204, 215
**Total Bilirubin (umol/L)**				
Baseline				
n	201		200	
Mean (SD)	4·4 (3·28)		4·3 (2·69)	
Median	4		4	
IQR (Q1, Q3)	(2, 5)		(2, 5)	
Min, Max	1, 32		1, 17	
Visit 3, Day 29				
n	197	197	191	191
Mean (SD)	4·2 (3·20)	-0·2 (2·66)	4·2 (2·49)	-0·1 (2·15)
Median	4	0	4	0
IQR (Q1, Q3)	(3, 5)	(-1, 1)	(2, 5)	(-1, 1)
Min, Max	0, 28	-19, 14	1, 19	-8, 7

SD, Standard Deviation; IQR, Interquartile range; Baseline defined as last non-missing assessment prior to first dose

Supplementary Table 10 Summary of HIV Viral Load (copies per mL) per Visit

Visit<br>Category	M72/AS01$_{E-4}$<br>> 200 copies per mL<br>n (%) [95% CI]		Placebo<br>> 200 copies per mL<br>n (%) [95% CI]
Baseline	N=201<br>3 (2)		N=200<br>3 (2)
Day 57	N=195<br>5 (3) [0·9, 5·6]	p-value 0·77	N=188<br>6 (3) [1·3, 6·5]
Day 210	N=192<br>13 (7) [3·8, 11]	p-value 0·26	N=176<br>7 (4) [1·8, 7·7]
Day 390/End of Study	N=194<br>15 (8) [4·6, 12·2]	p-value 0·42	N=181<br>10 (6) [2·8, 9·6]

HIV, human immunodeficiency virus; Baseline defined as last non-missing assessment prior to first dose
Percentages were based on number of participants with data at respective visit.
95% CIs of the percentages were computed using the Clopper-Pearson method with mid-p correction and p-value based on Fisher's exact test comparing the proportion of participants between groups·

Supplementary Table 11 Number and Percentage of Participants with $CD4^+$ T-Cell Counts <350 cells per µL Safety Population)

Visit	M72AS01$_{E-4}$ n (%) [95% CI]	Placebo n (%) [95% CI]
**Baseline**	N=201<br>7 (4) [1·5, 6·8]	N=200<br>5 (3) [0·9, 5·5]
**Visit 5 Day 57**	N=193<br>7 (4) [1·6, 7·0]	N=189<br>7 (4) [1·6, 7·2]
**Visit 6 Day 210**	N=191<br>8 (4) [2·0, 7·8]	N=175<br>9 (5) [2·5, 9·2]
**Visit 7 Day 390**	N=193<br>5 (3) [1·0, 5·7]	N=181<br>6 (3) [1·4, 6·8]

Baseline defined as last non-missing assessment prior to first dose
Percentages based on number of participants with data at respective visit
95% CIs of the percentages were computed using the Clopper-Pearson method with mid-p correction

Supplementary Table 12 Seropositivity Measured by ELISA by Timepoint (Per Protocol Population)

Visit Timepoint)<br>Statistics	M72/AS01$_{E-4}$<br>Antibody concentration<br>≥ 2·8 EU/mL<br>N=143<br>n (%), [CI]	Placebo<br>Antibody concentration<br>≥ 2·8 EU/mL<br>N=130<br>n (%), [CI]	p-value
Day 1	2 (1) [0·2, 4·5]	4 (3) [1·0, 7·3]	0·43
Day 29	128 (90) [83·7, 93·8]	4 (3) [1·0, 7·3]	< 0.0001
Day 57	143 (100) [97·9, 100]	4 (3) [1·0, 7·3]	< 0.0001
Day 210	141 (99) [95·5, 99·8]	4 (3) [1·0, 7·3]	< 0·0001
Day 390	140 (99) [95·4, 99·8]	4 (3) [1·0, 7·3]	< 0·0001

EU, ELISA enzyme-linked immunosorbent assay unit; N, number of participants with available results
Cut-off value for M72-specific antibody concentrations was 2·8 EU/mL
Seropositive against M72 defined as antibody concentration ≥ 2·8 EU/mL
Percentages computed using N as denominator
On Day 1 and Day 29 visits, samples were collected prior to trial intervention administration·
95% CIs of the percentages were computed using the Clopper-Pearson method with mid-p correction·
p-value based on Fisher's exact test comparing the percentages of participants between groups·

Supplementary Table 13 M72/AS01$_{E-4}$ Geometric Mean Antibody Concentration Results for the M72/AS01$_{E-4}$ Vaccine Group by Timepoint (Per Protocol Population)

	M72/AS01$_{E-4}$ Group<br>N=143<br>GMC (EU/mL) [95% CI]
Day 1	below LLOQ
Day 29	13·28 [10·99, 16·04]
Day 57	479·70 [421·79, 545·56]
Day 210	52·23 [44·83, 60·86]
Day 390*	32·43 [27·94, 37·65]

Below LLOQ,values were below lower limit of quantitation for M72-specific antibody concentrations of 2·8 EU/mL·
* N,142 at Day 390

Supplementary Table 14 Magnitude of M72 CD4⁺T Cell Positive IFN-γ and/or IL-2 Cytokine Response by Visit and Change from Baseline Day 1 (Per Protocol for Cellular Immunogenicity Population)

Visit	M72/AS01$_E$ (N=96) % IFN-γ and/or IL-2 expressing CD4$^+$ T cells	M72/AS01$_E$ (N=96) Change from Baseline	Placebo (N=32) % IFN-γ and/or IL-2 expressing CD4$^+$ T cells	Placebo (N=32) Change from Baseline
**Baseline Day 1**	n=96		n=32	
Mean (SD)	0.062 (0.506)		0.067 (0.167)	
Median	0.028		0.016	
IQR (Q1, Q3)	(0.011, 0.093)		(0.004, 0.068)	
p-value	0.16			
95% CI	(-0.041, 0.164)		(0.007, 0.127)	
Min, Max	-4.200, 1.790		-0.086, 0.769	
**Visit 5 Day 57**	n=96	n=96	n=31 [a]	n=31 [a]
Mean (SD)	0.534 (0.556)	0.473 (0.699)	0.014 (0.096)	-0.037 (0.213)
Median	0.383	0.296	0.014	-0.006
IQR (Q1, Q3)	(0.177, 0.663)	(0.139, 0.585)	(-0.015, 0.043)	(-0.018, 0.008)
p-value	<0.0001			
95% CI	(0.422, 0.647)	(0.331, 0.614)	(-0.021, 0.049)	(-0.115, 0.041)
Min, Max	0.036, 3.288	-0.694, 5.020	-0.400, 0.237	-1.169, 0.117
**Visit 7 Day 390**	n=96	n=96	n=32	n=32
(Mean SD)	0.339 (0.375)	0.277 (0.512)	0.047 (0.169)	-0.021 (0.194)
Median	0.266	0.175	0.011	-0.003
IQR (Q1, Q3)	(0.091, 0.464)	(0.075, 0.355)	(-0.002, 0.065)	(-0.022, 0.027)
p-value	<0.0001			
95% CI	(0.263, 0.415)	(0.173, 0.380)	(-0.014, 0.107)	(-0.091, 0.049)
Min, Max	-0.215, 2.709	-0.584, 4.608	-0.246, 0.855	-1.016, 0.289

Cell Type, CD4; Antigen, M72 peptides; CI, confidence interval; SD, standard deviation; IQR, Interquartile range

Baseline was defined as last non-missing assessment prior to first vaccination.

P-values based on the Wilcoxon-Mann Whitney test for pair-wise comparisons between treatment groups

a Note that 1 sample from the Day 57 timepoint was inadvertently not tested.

The percentage of participants with post-baseline CD4$^+$ T-cell response (based on M72-specific CD4$^+$ T cells expressing IFN-γ and/or IL-2) peaked at Day 57 and was sustained at Day 390 in the M72/AS01$_{E-4}$ group and was higher than in the placebo group at both Days 57 and 390.

Supplementary Table 15 Magnitude of M72 CD8+ T Cell Positive IFN-γ and/or IL-2 Cytokine Response by Visit and Change from Baseline Day 1 (Per Protocol for Cellular Immunogenicity Population)

Visit	M72/AS01E (N=96)		Placebo (N=32)	
	% IFN-γ and/or IL-2 expressing CD8+ T cells	Change from Baseline	% IFN-γ and/or IL-2 expressing CD8+ T cells	Change from Baseline
**Baseline Day 1**	n=96		n=32	
Mean (SD)	0.071 (0.248)		0.092 (0.353)	
Median	0.007		0.005	
IQR (Q1, Q3)	(0.000, 0.017)		(-0.006, 0.012)	
p-value	0.14			
95% CI	(0.021, 0.121)		(-0.036, 0.219)	
Min, Max	-0.306, 1.993		-0.045, 1.542	
**Visit 5 Day 57**	n=96	n=96	n=31*	n=31*
Mean (SD)	0.092 (0.272)	0.021 (0.164)	-0.007 (0.042)	-0.052 (0.276)
Median	0.009	0.003	-0.000	-0.005
IQR (Q1, Q3)	(0.001, 0.024)	(-0.005, 0.011)	(-0.012, 0.006)	(-0.014, 0.003)
p-value	0.0004			
95% CI	(0.037, 0.147)	(-0.012, 0.054)	(-0.022, 0.009)	(-0.153, 0.050)
Min, Max	-0.156, 1.531	-0.490, 1.214	-0.210, 0.039	-1.534, 0.045
**Visit 7 Day 390**	n=96	n=96	n=32	n=32
Mean (SD)	0.090 (0.328)	0.019 (0.112)	0.058 (0.371)	-0.033 (0.307)
Median	0.007	-0.000	0.003	-0.002
IQR (Q1, Q3)	(-0.002, 0.024)	(-0.008, 0.010)	(-0.008, 0.007)	(-0.010, 0.014)
p-value	0.04			
95% CI	(0.023, 0.156)	(-0.004, 0.042)	(-0.075, 0.192)	(-0.144, 0.077)
Min, Max	-0.230, 2.710	-0.254, 0.718	-0.311, 2.065	-1.635, 0.523

T Cell Type, CD8; Antigen, M72 peptides; CI, confidence interval; SD, standard deviation; IQR, Interquartile range
Baseline was defined as last non-missing assessment prior to first vaccination
P-values based on the Wilcoxon-Mann Whitney test for pair-wise comparisons between treatment groups
* One sample from the Day 57 timepoint was inadvertently not tested.

The median magnitude of M72-specific CD8+ T cells expressing IFN-γ and/or IL-2 was similar at baseline and at Day 57 (one month after Dose 2) suggesting the lack of induction of a CD8+ T cell response by the vaccine. The percentage of participants with post-baseline CD8+ T cell responses (based on M72-specific CD8+ T cells expressing IFN-γ and/or IL-2) was similar between the two groups (Supplementary Figure 3).

Supplementary Table 16 Percentages of Participants with Solicited AEs Based on IGRA Status at Baseline (Safety Population)

	M72/AS01$_{E-4}$		Placebo	
	**IGRA-Positive N=93**	**IGRA-Negative N=108**	**IGRA-Positive N=100**	**IGRA-Negative N=100**
	**n (%) [95% CI]**	**n (%) [95% CI]**	**n (%) [95% CI]**	**n (%) [95% CI]**
Pain, any	80 (86) [77·8, 92·0]	87 (81) [72·3, 87·2]	27 (27) [19·2, 36·7]	23 (24) [15·9, 32·6]
severe	17 (18) [11·4, 27·1]	15 (14) [8·3, 21·4]	2 (2) [0·3, 6·5]	0 [0·0, 0·02]
Redness, any	36 (39) [29·2, 48·9]	31 (29) [20·8, 37·8]	9 (9) [4·5, 16·0]	10 (10) [5·3, 17·4]
severe	0 [0·0, 0·02]	0 [0·0, 0·02]	0 [0·0, 0·02]	0 [0·0, 0·02]
Swelling, any	50 (54) [44·1, 64·3]	33 (31) [22·4, 39·7]	9 (9) [4·5, 16·0]	8 (8) [3·9, 14·9]
severe	0 [0·0, 0·02]	0 [0·0, 0·02]	0 [0·0, 0·02]	0 [0·0, 0·02]
Fever, any	51 (55) [44·7, 64·7]	36 (33) [24·9, 42·6]	29 (29) [21·0, 38·8]	15 (15) [9·2, 23·5]
severe	3 (3) [0·8, 8·5]	0 [0·0, 0·02]	1 (1) [0·1, 4·9]	1 (1) [0·1, 4·9]
Headache, any	65 (70) [60·0,78·6]	67 (62) [52·6, 70·8]	42 (42) [33·0, 52·3]	36 (37) [27·6, 46·6]
severe	15 (16) [9·7, 24·7]	6 (6) [2·3, 11·2]	6 (6) [2·5, 12·2]	1 (1) [0·1, 4·9]
Fatigue, any	61 (66) [55·5, 74·7]	58 (54) [44·3, 62·9]	40 (40) [31·1, 50·3]	34 (35) [25·8, 44·5]
severe	10 (11) [5·6,18·3]	3 (3) [0·7, 7·4]	2 (2) [0·3, 6·5]	2 (2) [0·3, 6·6]
GI symptoms, any	26 (28) [19·6, 37·7]	30 (28) [20·0, 36·8]	23 (23) [15·7, 32·3]	15 (15) [9·2, 23·5]
severe	3 (3) [0·8, 8·5]	1 (1) [0·0, 4·5]	0 [0·0, 0·02]	0 [0·0, 0·02]
Myalgia, any	49 (53) [42·5, 62·7]	47 (44) [34·4, 53·0]	25 (25) [17·4, 34·5]	20 (20) [13·3, 29·2]
severe	10 (11) [5·6, 18·3]	8 (7) [3.5, 13.6]	1 (1) [0·1, 4·9]	1 (1) [0·1, 4·9]

Data reported as collected on the diary card from Days 1 to 7 after dosing (highest grade experienced for a given AE was recorded).
95% CI of the percentage was based on the binomial Clopper-Pearson method with mid-p correction
N, number of participants in the Safety Population; n, number of participants with event
Any injection area AE included participants with pain, redness > 0 mm, or swelling > 0 mm
Redness and Swelling: Any > 0 mm; Severe (grade 3) ≥ 100 mm
Any general body symptom included participants with any fever (≥ 37.5°C), headache, fatigue (tiredness), gastrointestinal symptoms (stomach problems), myalgia (muscle pain).
Any fever refers to temperature ≥ 37.5°C; Severe fever refers to ≥ 39.3 to < 40.0°C

There was an overall trend in which vaccine recipients with positive IGRA status at baseline had somewhat higher percentages of solicited AEs than those with negative IGRA status. These differences were most notable with any injection site swelling and any fever, along with severe headache and severe fatigue.
Among participants in the placebo group, baseline IGRA status did not appear to have any effect on the frequency of solicited AEs.

PREGNANCY

Although measures were taken to avoid pregnancies during the 13-month duration of the trial, nine women became pregnant, and six infants had no notable birth complications· Three women experienced spontaneous abortion, two in the M72 group, and one in the placebo group· The two women in the M72/AS01$_{E\text{-}4}$ group had histories of previous abortions or fetal death· All of the abortions were judged to be unrelated to trial intervention·

Supplementary Figure 2 Percentage of Participants Reporting Solicited Adverse Events

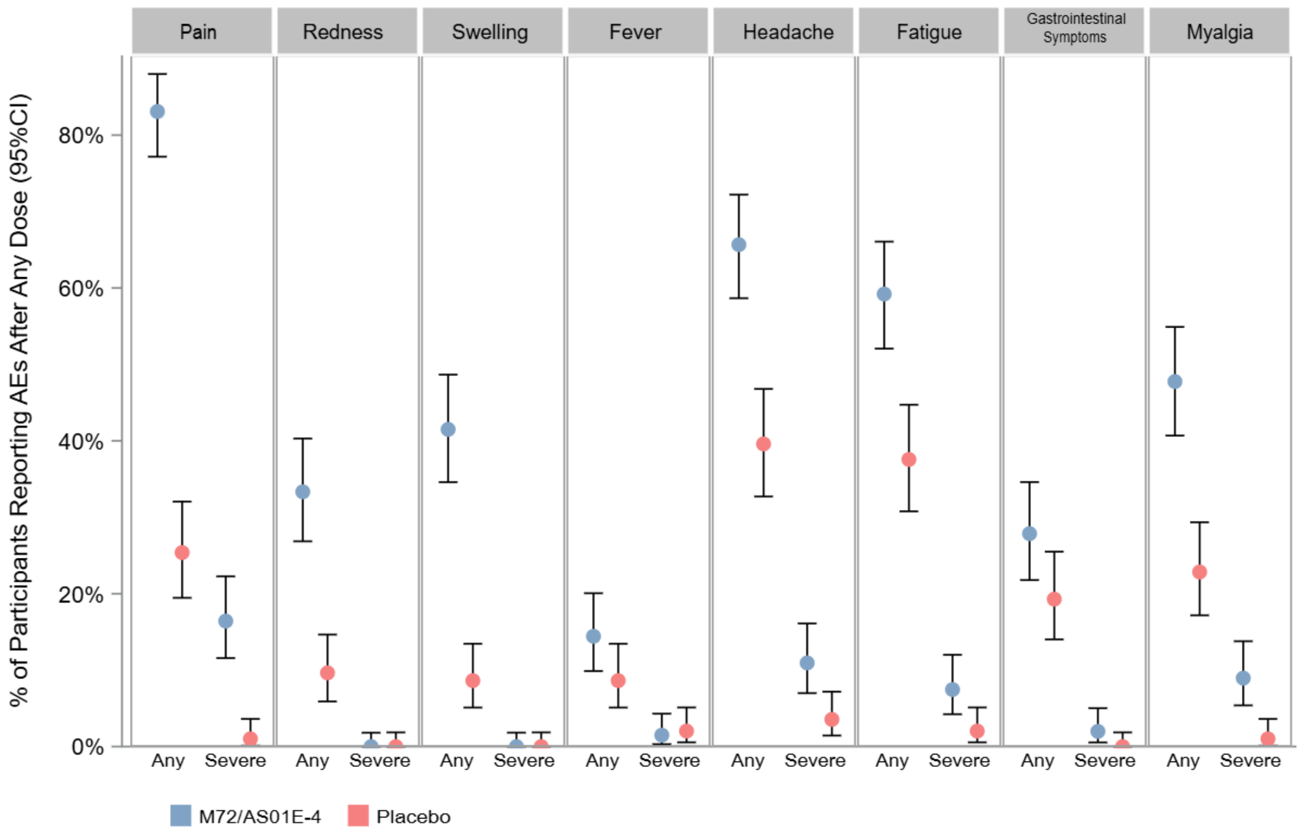

legend
Any AE refers to the percentage of participants reporting solicited AEs after Dose 1 or Dose 2, (i.e., overall reported after Dose 1 and/or Dose 2) using the highest grading of each AE reported (Error bars represent 95% CIs).
AE, Adverse Event
Redness and swelling were graded based on the largest diameter measured in mm: Mild, 25 to < 50 mm; Moderate, ≥ 50 to < 100 mm; Severe ≥ 100 mm.
Fever was graded as mild, 38·0 to < 38·6°C; moderate ≥ 38·6 to < 39·3°C; severe ≥ 39·3 to < 40·0°C). All other solicited AEs were graded as mild (Grade 1, not interfering with normal daily activities), moderate (Grade 2, interfering with normal daily activities), severe (Grade 3, preventing normal daily activities), or potentially life-threatening (Grade 4).
Note that the first 2 bars represent Any AE (regardless of severity grading) and the second 2 bars represent AEs graded as severe.
There were no Grade 4 AEs reported.

Supplementary Figure 3 Longitudinal Magnitude of CD4+ T-cell Cytokine IFN-γ and/or IL-2 Response by Participant (Per Protocol for Cellular Immunogenicity Population)

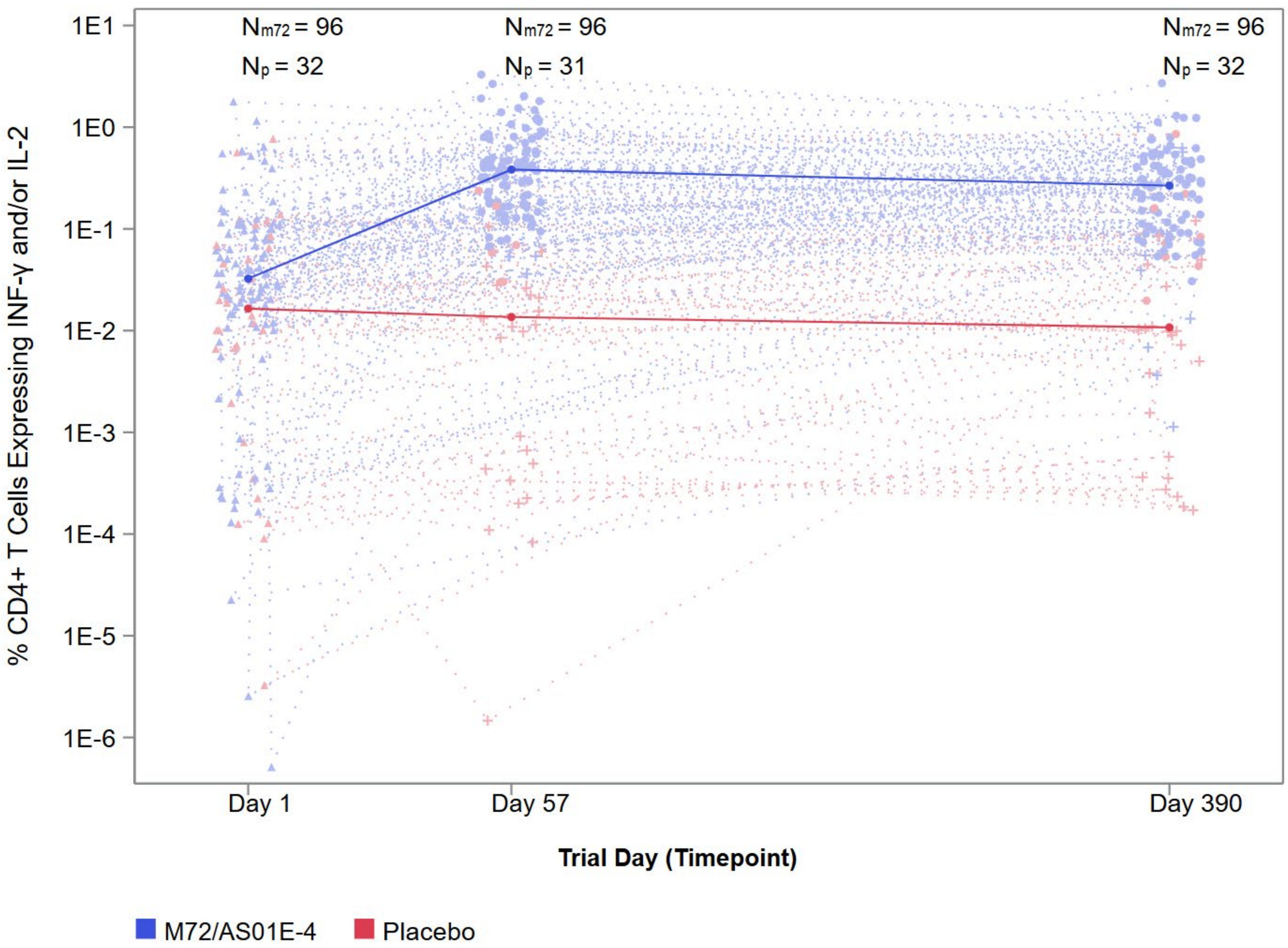

Supplementary Figure 4 Longitudinal Magnitude of CD8$^+$ T-cell Cytokine IFN-γ and/or IL-2 Response by Participant (Per Protocol for Cellular Immunogenicity Population)

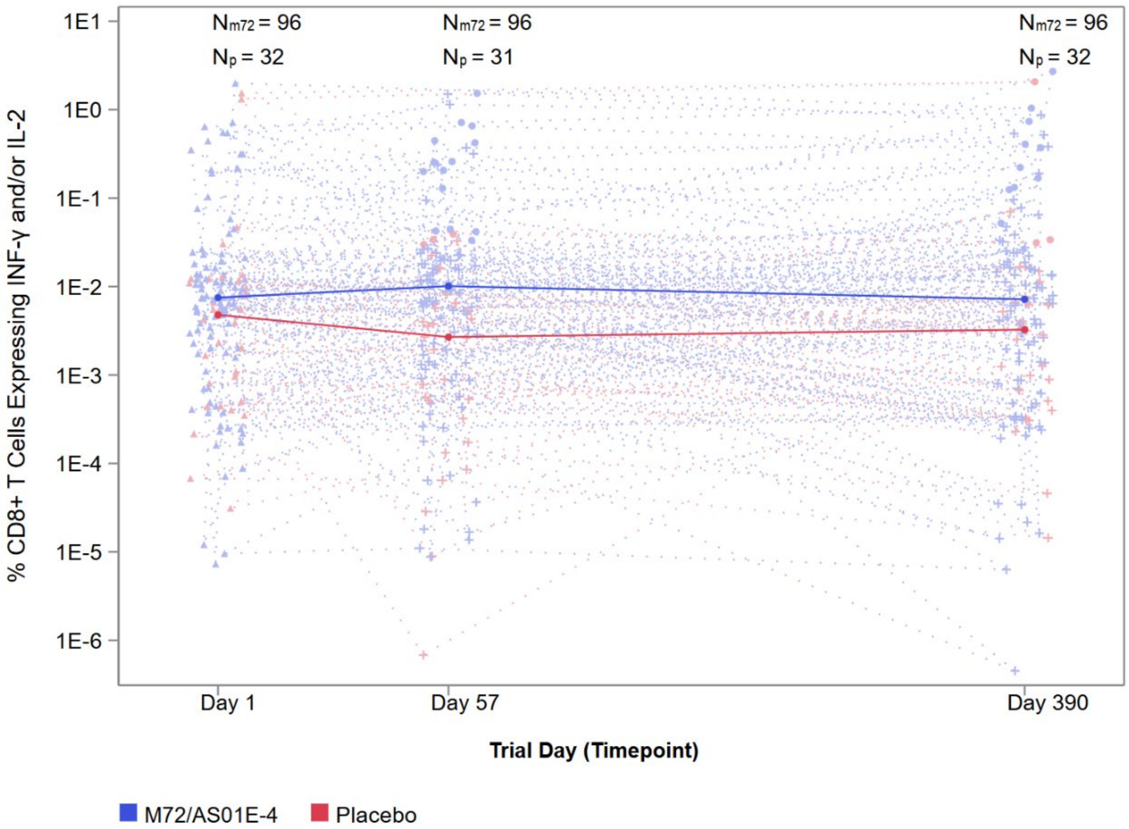

legend

Dashed lines connect individual participants· Solid lines indicate median magnitude per trial arm·

M72/AS01$_{E-4}$ responder status was based on cytokine combination (IFN-γ and/or IL-2) and determined for each participant study group, T cell type, and visit·

M72/AS01$_{E-4}$ responders are indicated by means of a closed circle; non-responders are indicated by means of a cross; missing responder status is indicated by means of a triangle· IFN-γ, Interferon-gamma; IL-2, Interleukin-2

text

The percentages of participants with post-baseline CD8$^+$ T cell responses based on M72-specific CD8$^+$ T cells expressing IFN-γ and/or IL-2) were similar between groups: 15% (14/96) in the M72/AS01$_{E-4}$ group vs 10% (3/31) in the placebo group (p=0·76), at Day 57, and at Day 390, 10% (10/96) in the M72/AS01$_{E-4}$ group vs 9% (3/32) in the placebo group (p=1·00).

Supplementary Figure 5 M72-Specific CD4^{+} T-cell Polyfunctional Plots (Per Protocol for Cellular Immunogenicity Population)

5a.

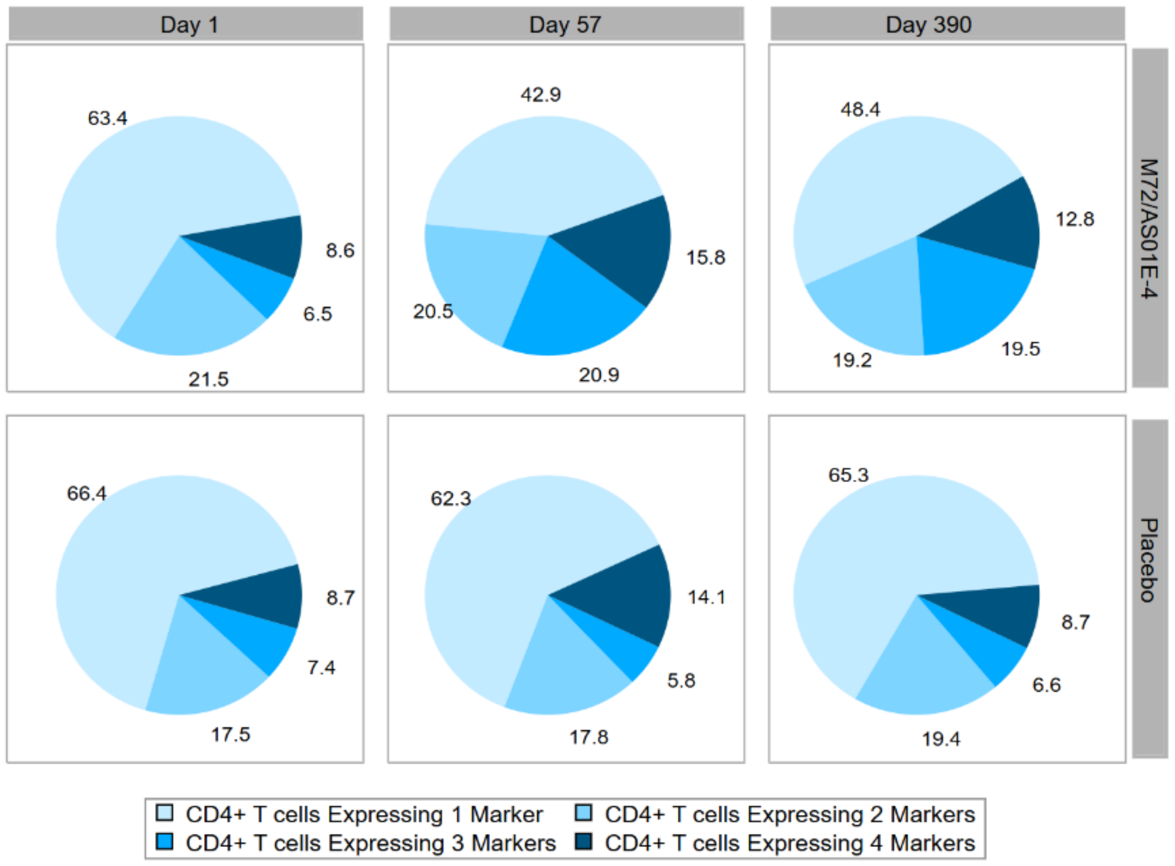

legend

Mean proportion of M72-specific CD4^{+} T cells expressing 1, 2, 3 or 4 functional markers at Day 1, Day 57 and Day 390 for vaccine recipients (top row) and placebo recipients (bottom row)

5b.

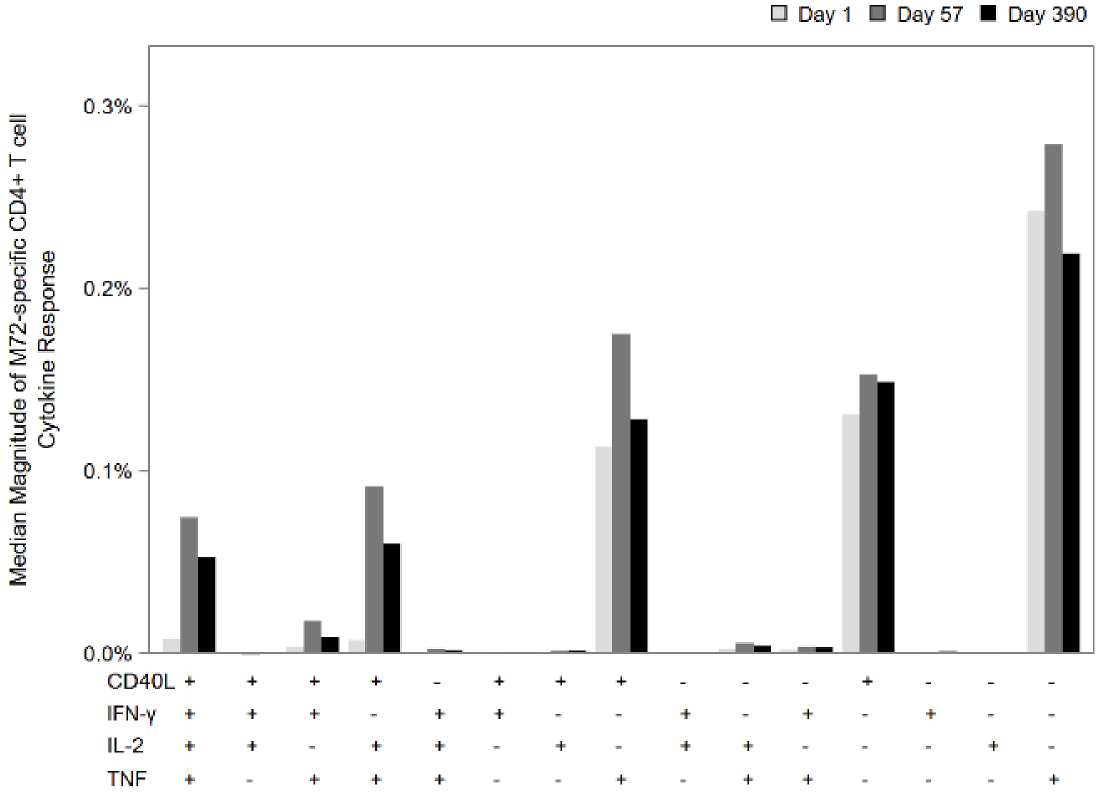

legend

Median Magnitude of M72 antigen-specific polyfunctional CD4$^+$ T cells for each functional marker combination as shown below the X-axis, at Day 1, Day 57, and Day 390 for vaccine recipients·

+=positive response

-=negative response

CD40L=CD40 ligand

IFN-γ=Interferon-gamma

IL-2=Interleukin-2

TNF=Tumor Necrosis Factor

Figure 5 text:

To assess the polyfunctionality of M72-specific CD4$^+$ T cells, co-expression of IFN-γ, IL2, TNF and CD40L following M72 stimulation of CD4$^+$ T cells was measured by ICS.A) shows that the proportion of triple and quadruple-positive M72-specific CD4$^+$ T cells increased from Day 1 to Day 57, then slightly contracted by Day 390 in vaccine recipients, while no changes were apparent over time in the placebo group. The distribution of the different marker combinations as described under the X-axis for the M72/AS01$_{E-4}$ group. B) shows the pronounced increase in CD40L/TNF/IL-2- and CD40L/TNF/IFN-γ-expressing triple-positive as well as the quadruple-positive populations following vaccination·

In line with the insignificant increase in magnitude of CD8$^+$ T-cell responses following vaccination, polyfunctionality for this subset did not change over the course of the post-vaccination timepoints.

Supplementary Table 17 Geometric Mean Antibody Concentrations by IGRA for Participants in the M72/AS01$_{E-4}$ Group (Per Protocol for Cellular Immunogenicity Population)

	IGRA-positive N=63	IGRA-negative N=80
Day 1	below LLOQ	below LLOQ
Day 29	19·75 [14·91, 26·17]	9·71 [7·66, 12·32]
Day 57	559·49 [461·75, 677·93]	424·95 [357·74, 504·80]
Day 210	71·99 [58·80, 88·13]	40·57 [32·92, 50·01]
Day 390	43·99 [35·98, 53·79]	25·61 [20·91, 31·37]

Below LLOQ=values were below lower limit of quantitation for M72-specific antibody concentrations of 2·8 EU/mL

Among the 143 participants in the PP Population of the M72/AS01$_{E-4}$ group, 63 participants were IGRA-positive at baseline, and 80 participants were IGRA-negative at baseline· Among participants in the M72/AS01$_{E-4}$ group, those who were IGRA-positive at baseline had GMCs notably higher than those who were IGRA-negative at baseline at Days 29, 210 and 390.· For all post-baseline values, GMCs in the IGRA-positive group were higher than in the IGRA-negative group, with peak values at Day 57 of 559·49 EU/mL and 424·95 EU/mL, respectively·

Supplementary Figure 6 Geometric Mean Antibody Concentrations Overall and by IGRA Status at Baseline (Per Protocol Population)

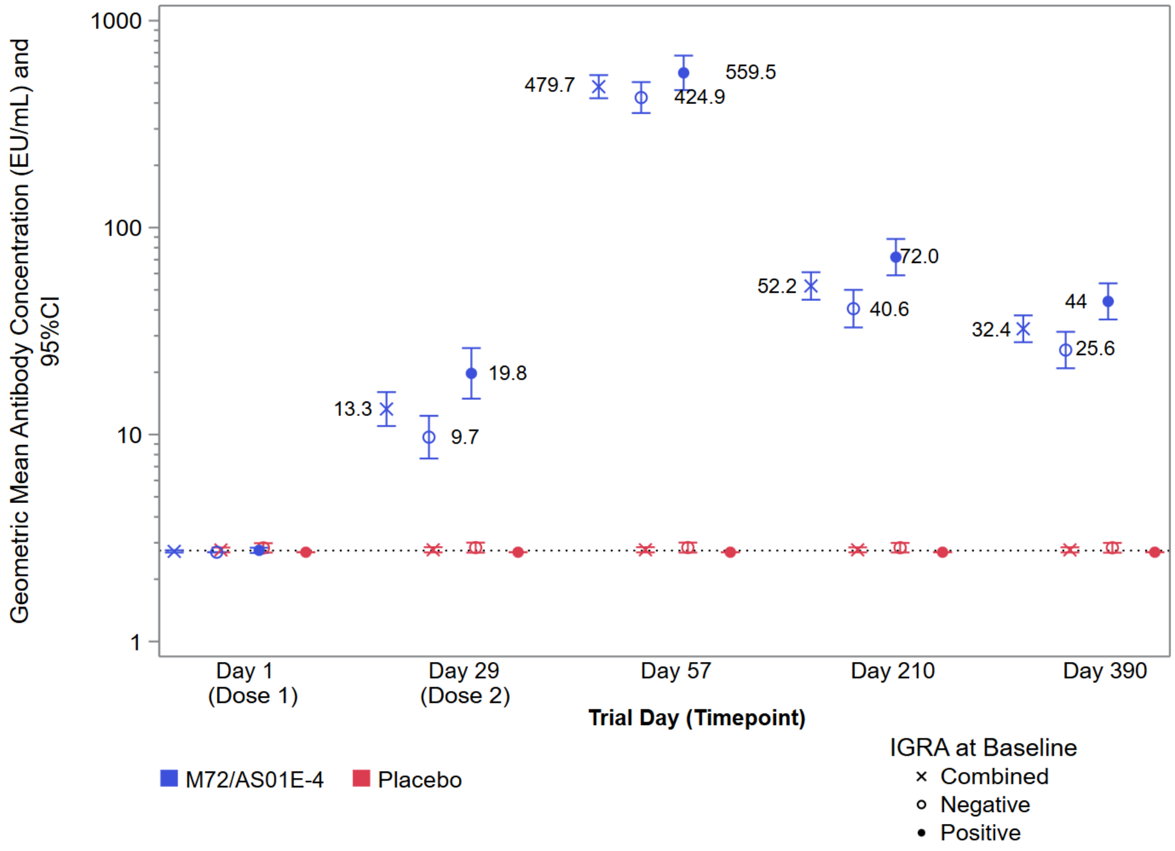

legend

Geometric mean concentration of M72-specific IgG antibody in ELISA units per milliliter. Error bars represent 95% CIs.

Combined, Interferon-Gamma Release Assay (IGRA) positive and negative at baseline; Negative, IGRA-negative at baseline; Positive, IGRA-positive at baseline

Supplementary Figure 7 Magnitude of M72-Specific CD4+ T-cell IFN-γ and/or IL-2 Response, by IGRA Status at Baseline, M72/AS01$_{E-4}$ Group Only (Per Protocol for Cellular Immunogenicity Population)

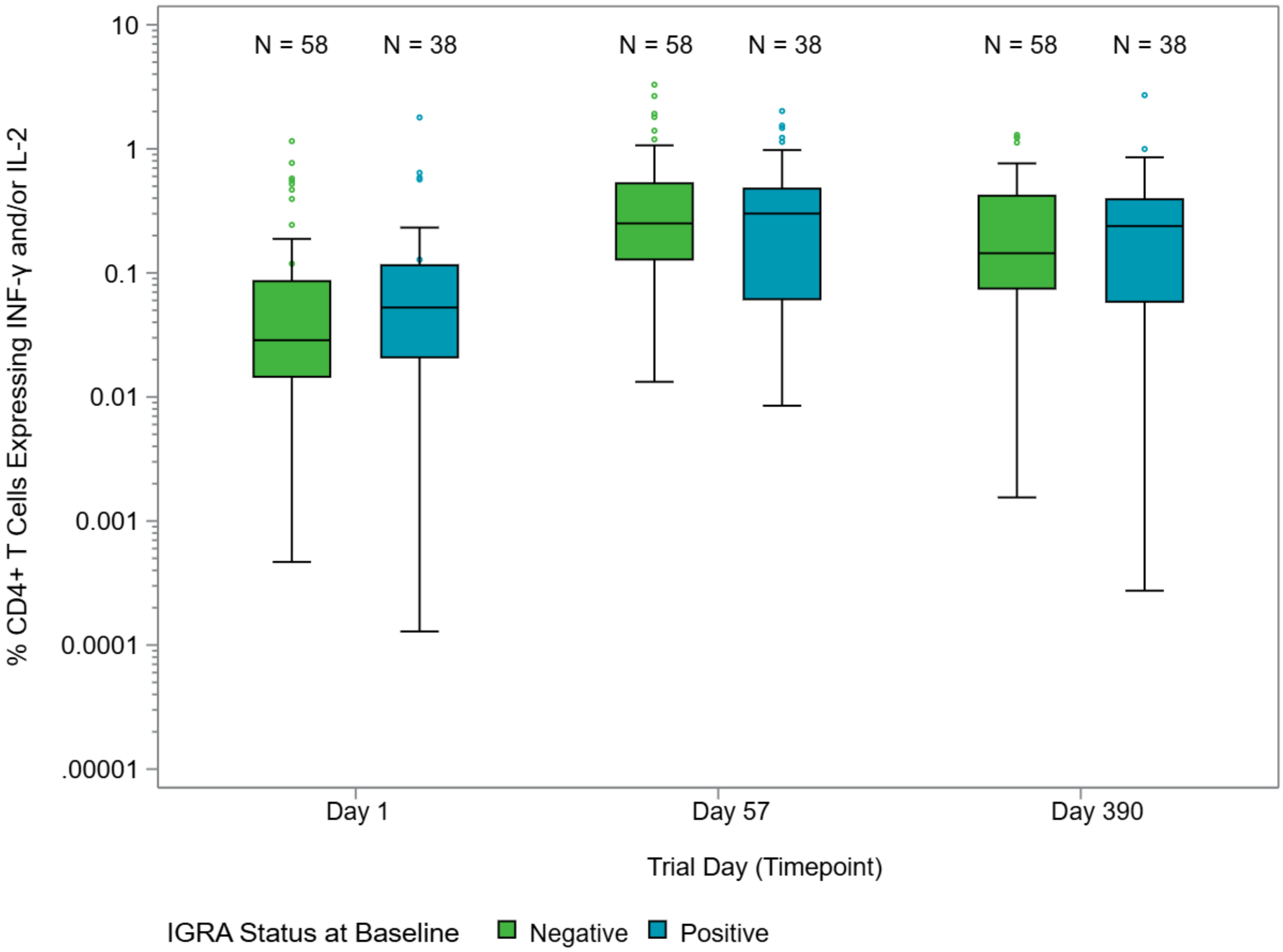

legend

Whiskers represent the observed lower and upper inner fences where the inner fences are defined as Q1 – 1.5IQR (lower) and Q3 + 1.5IQR (upper)

Box ranges represent interquartile range

Middle line represents median· values

IFN-γ, Interferon- gamma; IL-2, Interleukin-2; IGRA, Interferon-Gamma Release Assay

Supplementary Figure 8 Longitudinal Magnitude of M72-Specific CD4$^+$ T-cell IFN-γ and/or IL-2 Response by IGRA Status at Baseline (Per Protocol for Cellular Immunogenicity Population)

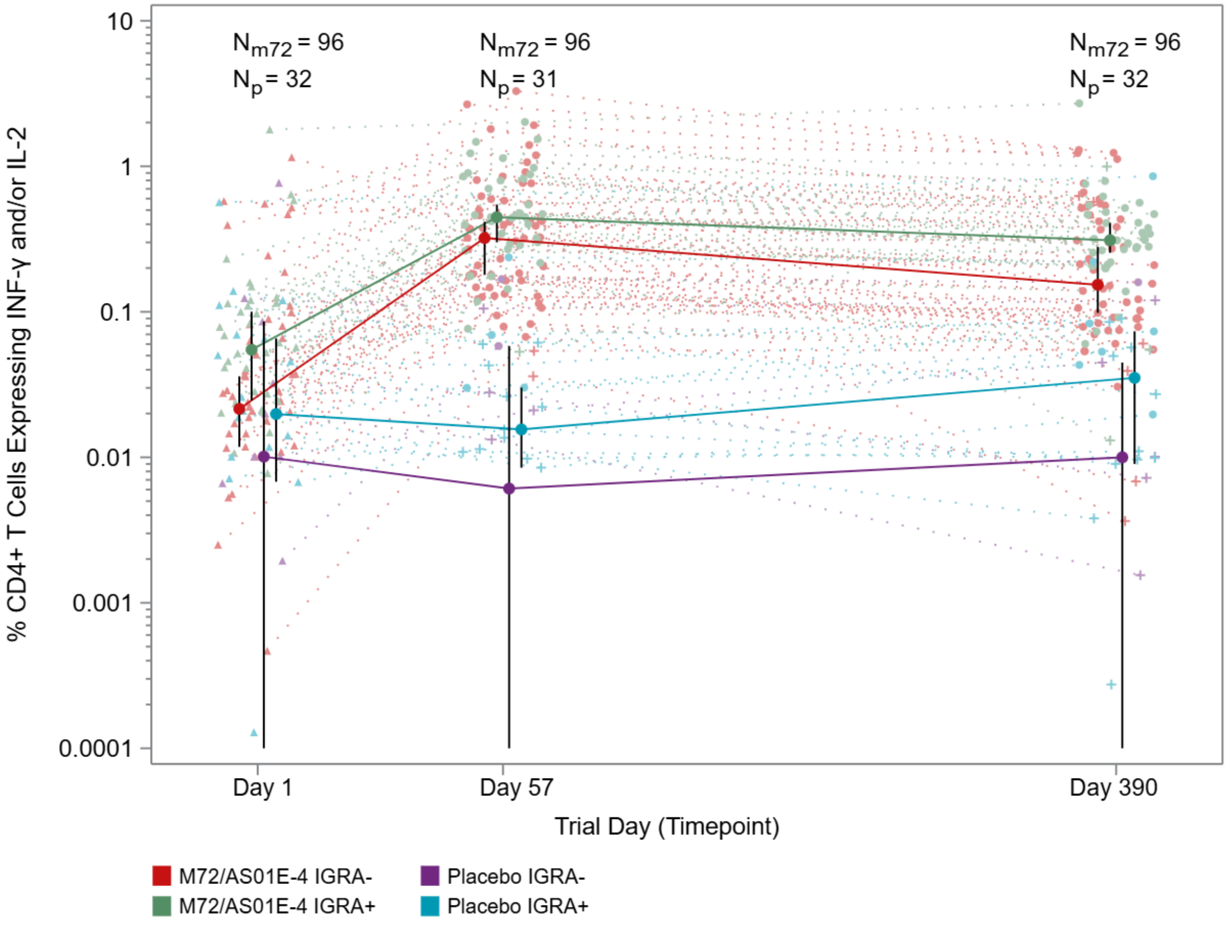

legend

N_{m72}, total number of participants in the M72/AS01$_{E-4}$ group. N_p, total number of participants in the placebo group. Dashed lines connect individual participants. Solid lines connect median magnitude of M72- specific CD4$^+$ responses at different timepoints per trial arm. M72-specific responder status is based on CD4$^+$ T cells expressing IFN-γ and/or IL-2 and determined for each participant, stimulation agent, and visit. M72-specific responders are indicated with a closed circle; M72/AS01$_{E-4}$ non-responders are indicated by a cross; pre-vaccination status is indicated with a triangle.

Bolded lines and points indicate median, vertical lines indicate the 95% confidence intervals of the medians

Supplementary Table 18 Cell-Mediated Immune Response: Percentage of Participants with Post-Baseline M72-Specific CD4+ T-Cell Response by IGRA Status at Baseline (Per Protocol for Cellular Immunogenicity Population)

	Day 57		Day 390	
**Group**	**IGRA-positive at baseline % [95% CI]**	**IGRA-negative at baseline % [95% CI]**	**IGRA-positive at baseline % [95% CI]**	**IGRA-negative at baseline % [95% CI]**
	N=38	N=58	N=38	N=58
M72	97·4 [87·7, 99·9]	94·8 [86·6, 98.7]	92·1 [80·0, 98·0]	86·2 [75·5, 93·4]
	N=19	N=12	N=20	N=12
Placebo	21·1 [7·1, 43·3]	16·7 [2·9, 45·1]	35·0 [16·8, 57·3]	8·3 [0·4, 34·8]

N, total number of participants in the Per Protocol for Cellular Immunogenicity population with data available to determine M72/AS01E-4 responder status; %, percentage of participants with positive response
p-value based on Fisher's exact test comparing the percentage of responders between treatment groups
Responder status is based on positive IFN-γ and/or IL-2 cytokine combination post-baseline, and determined for each participant, T cell type, treatment group, and visit

Supplementary Figure 9 Longitudinal Magnitude of CD8$^+$ T-Cell IFN-γ and/or IL-2 Response by IGRA status at Baseline (Per Protocol for Cellular Immunogenicity Population)

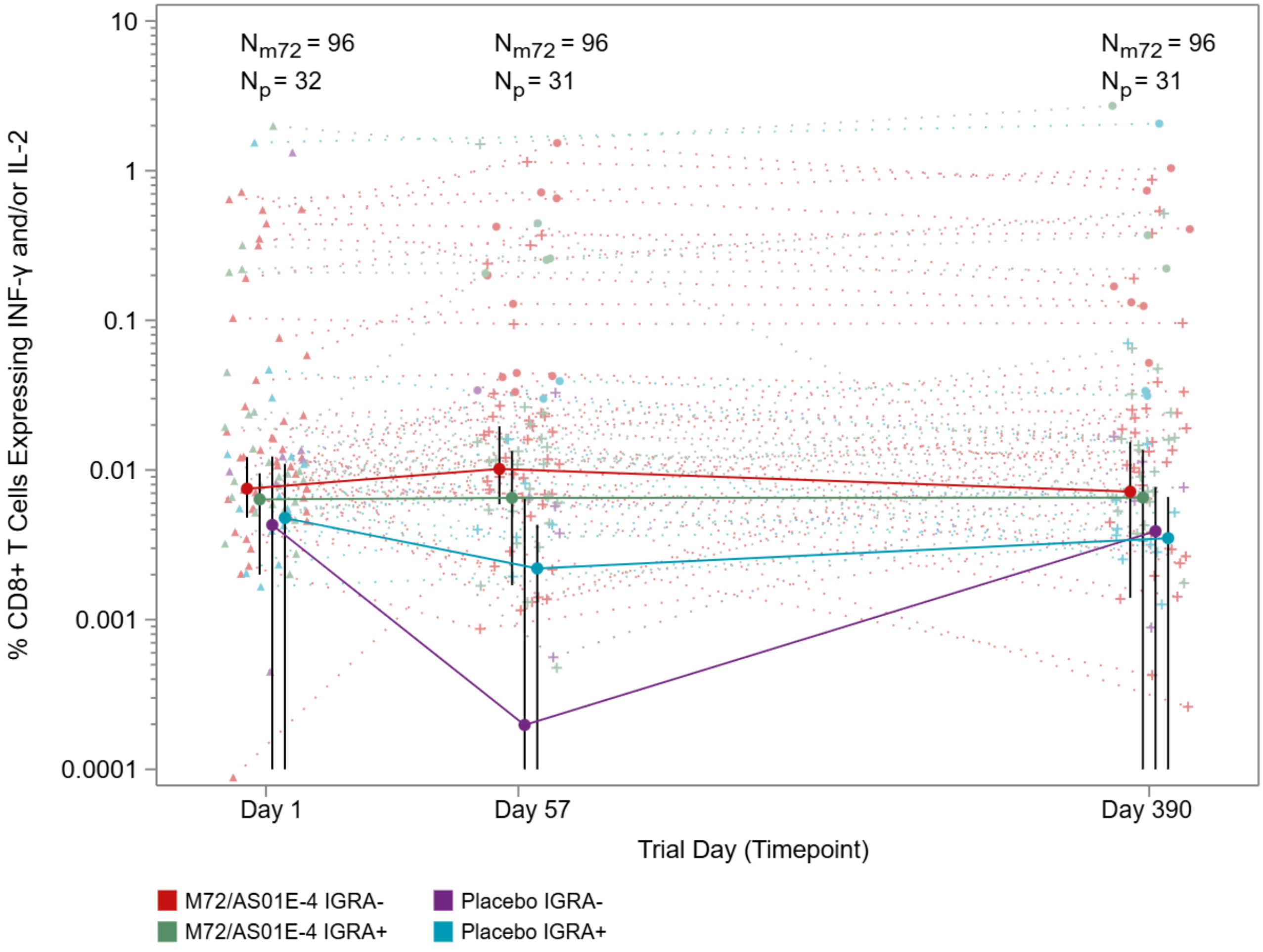

legend

N_{m72}, total number of participants in the M72/AS01$_{E\text{-}4}$ group. N_p, total number of participants in the placebo group. Dashed lines connect individual participants. Solid lines connect median magnitude of M72- specific CD4$^+$ responses at different timepoints per trial arm. M72-specific responder status is based on CD4$^+$ T cells expressing IFN-γ and/or IL-2 and determined for each participant, stimulation agent, and visit. M72-specific responders are indicated with a closed circle; M72/AS01$_{E\text{-}4}$ non-responders are indicated by a cross; pre-vaccination status is indicated with a triangle.

Bolded lines and points indicate median; vertical lines indicate the 95% confidence intervals of the medians

text

There were no apparent differences in the median magnitude of CD8$^+$ T cells expressing IFN-γ and/or IL-2 among vaccine recipients who had a positive or negative IGRA status at baseline.

Supplementary Table 19 Magnitude of M72 CD4$^+$T Cell Positive IFN-γ and/or IL-2 Cytokine Response by Visit and Change from Baseline Day 1 among IGRA Positive Participants at Baseline (Per Protocol for Cellular Immunogenicity Population)

Visit	M72/AS01$_E$ (N=38) % IFN-γ and/or IL-2 expressing CD4$^+$ T cells	M72/AS01$_E$ (N=38) Change from Baseline	Placebo (N=20) % IFN-γ and/or IL-2 expressing CD4$^+$ T cells	Placebo (N=20) Change from Baseline
**Baseline Day 1**	n=38		n=20	
Mean (SD)	0.026 (0.768)		0.058 (0.131)	
Median	0.055		0.020	
IQR (Q1, Q3)	(0.021, 0.115)		(0.007, 0.068)	
p-value	0.0526			
95% CI	(-0.226, 0.279)		(-0.003, 0.119)	
Min, Max	-4.200, 1.790		-0.086, 0.566	
**Visit 5 Day 57**	n=38	n=38	n=19	n=19
Mean (SD)	0.574 (0.442)	0.547 (0.828)	0.029 (0.058)	-0.002 (0.040)
Median	0.447	0.367	0.016	-0.006
IQR (Q1, Q3)	(0.287, 0.819)	(0.190, 0.589)	(0.008, 0.043)	(-0.016, 0.008)
p-value	<0.0001			
95% CI	(0.428, 0.719)	(0.275, 0.819)	(0.001, 0.057)	(-0.021, 0.017)
Min, Max	0.053, 2.017	-0.106, 5.020	-0.042, 0.237	-0.079, 0.117
**Visit 7 Day 390**	n=38	n=38	n=19	n=19
(Mean SD)	0.406 (0.437)	0.380 (0.730)	0.075 (0.196)	0.017 (0.083)
Median	0.310	0.228	0.035	0.001
IQR (Q1, Q3)	(0.209, 0.469)	(0.143, 0.367)	(0.009, 0.078)	(-0.018, 0.027)
p-value	<0.0001			
95% CI	(0.263, 0.550)	(0.140, 0.620)	(-0.017, 0.167)	(-0.021, 0.056)
Min, Max	0.013, 2.709	0.002, 4.608	-0.154, 0.855	-0.123, 0.289

Cell Type, CD4; Antigen, M72 peptides; CI, confidence interval; SD, standard deviation; IQR, Interquartile range

Baseline was defined as last non-missing assessment prior to first vaccination.

P-values based on the Wilcoxon-Mann Whitney test for pair-wise comparisons between treatment groups

Supplementary Table 20Magnitude of M72 CD4^{+}T Cell Positive IFN-γ and/or IL-2 Cytokine Response by Visit and Change from Baseline Day 1 among IGRA Negative Participants at Baseline (Per Protocol for Cellular Immunogenicity Population)

	M72/AS01$_E$ (N=58)		Placebo (N=12)	
**Visit**	**% IFN-γ and/or IL-2 expressing CD4^{+} T cells**	**Change from Baseline**	**% IFN-γ and/or IL-2 expressing CD4^{+} T cells**	**Change from Baseline**
**Baseline Day 1**	n=58		n=12	
Mean (SD)	0.085 (0.205)		0.083 (0.221)	
Median	0.022		0.010	
IQR (Q1, Q3)	(0.003, 0.063)		(-0.005, 0.059)	
p-value	0.4498			
95% CI	(0.031, 0.139)		(-0.057, 0.223)	
Min, Max	-0.161, 1.154		-0.033, 0.769	
**Visit 5 Day 57**	n=58	n=58	n=12	n=12
Mean (SD)	0.509 (0.622)	0.424 (0.602)	-0.009 (0.137)	-0.092 (0.341)
Median	0.321	0.217	0.006	-0.006
IQR (Q1, Q3)	(0.147, 0.581)	(0.128, 0.581)	(-0.026, 0.043)	(-0.018, 0.007)
p-value	<0.0001			
95% CI	(0.345, 0.672)	(0.265, 0.582)	(-0.096, 0.078)	(-0.309, 0.124)
Min, Max	0.036, 3.288	-0.694, 3.178	-0.400, 0.168	-1.169, 0.082
**Visit 7 Day 390**	n=58	n=58	n=12	n=12
(Mean SD)	0.294 (0.325)	0.209 (0.280)	-0.001 (0.099)	-0.084 (0.295)
Median	0.153	0.121	-0.001	-0.004
IQR (Q1, Q3)	(0.079, 0.433)	(0.058, 0.285)	(-0.020, 0.027)	(-0.022, 0.015)
p-value	<0.0001			
95% CI	(0.209, 0.380)	(0.136, 0.283)	(-0.064, 0.062)	(-0.272, 0.104)
Min, Max	-0.215, 1.295	-0.584, 1.213	-0.246, 0.160	-1.016, 0.074

Cell Type, CD4; Antigen, M72 peptides; CI, confidence interval; SD, standard deviation; IQR, Interquartile range

Baseline was defined as last non-missing assessment prior to first vaccination.

P-values based on the Wilcoxon-Mann Whitney test for pair-wise comparisons between treatment groups
